# The effects of low-carbohydrate diets on cardiovascular risk factors: A meta-analysis

**DOI:** 10.1371/journal.pone.0225348

**Published:** 2020-01-14

**Authors:** Tingting Dong, Man Guo, Peiyue Zhang, Guogang Sun, Bo Chen

**Affiliations:** 1 Human Anatomy Histology and Embryology, Southwest Medical University, Luzhou, PR China; 2 Department of Diabetes and Endocrinology, Affiliated Hospital of Southwest Medical University, Luzhou, PR China; University of Mississippi Medical Center, UNITED STATES

## Abstract

**Background:**

Low-carbohydrate diets are associated with cardiovascular risk factors; however, the results of different studies are inconsistent.

**Purpose:**

The aim of this meta-analysis was to assess the relationship between low-carbohydrate diets and cardiovascular risk factors.

**Method:**

Four electronic databases (PubMed, Embase, Medline, and the Cochrane Library) were searched from their inception to November 2018. We collected data from 12 randomized trials on low-carbohydrate diets including total cholesterol, high-density lipoprotein cholesterol (HDL-C), low-density lipoprotein cholesterol (LDL-C), triglycerides, and blood pressure levels, as well as weight as the endpoints. The average difference (MD) was used as the index to measure the effect of a low-carbohydrate diet on cardiovascular risk factors with a fixed-effects model or random-effects model. The analysis was further stratified by factors that might affect the results of the intervention.

**Results:**

From 1292 studies identified in the initial search results, 12 randomized studies were included in the final analysis, which showed that a low-carbohydrate diet was associated with a decrease in triglyceride levels of -0.15mmol/l (95% confidence interval -0.23 to -0.07). Low-carbohydrate diet interventions lasting less than 6 months were associated with a decrease of -0.23mmol/l (95% confidence interval -0.32 to -0.15), while those lasting 12–23 months were associated with a decrease of -0.17mmol/l (95% confidence interval -0.32 to -0.01). The change in the body weight in the observation groups was -1.58kg (95% confidence interval -1.58 to -0.75); with for less than 6 months of intervention,this change was -1.14 kg (95% confidence interval -1.65 to -0.63),and with for 6–11 months of intervention, this change was -1.73kg (95% confidence interval -2.7 to -0.76). The change in the systolic blood pressure of the observation group was -1.41mmHg (95% confidence interval—2.26 to -0.56); the change in diastolic blood pressure was -1.71mmHg (95% confidence interval—2.36 to -1.06); the change in plasma HDL-C levels was 0.1mmHg (95% confidence interval 0.08 to 0.12); and the change in serum total cholesterol was 0.13mmol/l (95% confidence interval 0.08 to 0.19). The plasma LDL-C level increased by 0.11mmol/l (95% confidence interval 0.02 to 0.19), and the fasting blood glucose level changed 0.03mmol/l (95% confidence interval -0.05 to 0.12),which was not significant.

**Conclusions:**

This meta-analysis confirms that low-carbohydrate diets have a beneficial effect on cardiovascular risk factors but that the long-term effects on cardiovascular risk factors require further research.

## Introduction

According to statistics from the World Health Organization, 17 million people die of cardiovascular disease every year [1], and 80% of cardiovascular disease deaths occur in developing countries [2]. The main risk factors for cardiovascular diseases include obesity, abnormal blood lipid profiles, and unreasonable diets; among these, abnormal blood lipid profiles increase the risk of hypertension, coronary heart disease, metabolic syndrome and type 2 diabetes and increase the morbidity and mortality of individuals with cardiovascular diseases[3–5].

Diets with high levels of carbohydrates, especially refined or high glycemic index carbohydrates, also appear to be associated with hypertension, coronary heart disease, obesity, type 2 diabetes, metabolic syndrome and increased risk of mortality.[6–8]. In recent years, the public has become increasingly aware of this problem and its impact on global health. This problem is speculated to be caused by excessive energy intake, low energy consumption, or both. Furthermore, an increasing number of studies have focused on the association between cardiovascular diseases in different diets, and the debate about which diet is more beneficial for protection against cardiovascular diseases is intensifying. Low-carbohydrate diets, which limit carbohydrates and increase the percentage of fat or protein, are a popular weight-loss strategy; however, their cardiovascular effects are unknown. Prospective cohort studies have produced conflicting results regarding the association between low-carbohydrate dietary patterns and the risk of cardiovascular disease [9,10]. Studies have shown that low-carbohydrate diets are effective for losing weight, improving cardiovascular risk factors and preventing or treating diabetes [11–13]. However, Lagiou analyzed data from large cohorts and showed that long-term low-carbohydrate diets increased the effects of cardiovascular risk factors and shortened lifespan [14].

Therefore, we conducted a systematic meta-analysis to determine whether low-carbohydrate diets had any beneficial or detrimental effects on cardiovascular risk factors.

## Methods

### Data sources and searches

This meta-analysis is reported in accordance with the Preferred Reporting Items for Systematic Reviews and Meta-Analyses (PRISMA) statement (S1 Table). We searched the PubMed, Embase, Medline and Cochrane Central Register of Controlled Trials databases from their inception up to November 2018. The full electronic search strategy is detailed in S2 Table. The aim of this study was to determine the effect of a low-carbohydrate diet on cardiovascular risk factors using randomized controlled studies. According to the characteristics of the different databases, a combination of MeSH words and free words was used for retrieval using the query ‘diet’ + ‘trial’ + ‘lowcarbohydrate’. The search was restricted to human studies. Only articles published in English were included. The reference lists of the original studies were manually searched to retrieve all relevant literature.

### Inclusion criteria

The inclusion criteria were as follows: (1) the study was a randomized clinical trial; (2) the study population was at least 18 years old and had no specific diseases; (3) the trial had at least a 3-month follow-up period after the initiation of the diet; (4) the proportion of carbohydrates in the low-carbohydrate diet was less than 40%; (5) the most recent and complete study was used if data from the same population were published more than once.

### Exclusion criteria

Studies including any of the following were excluded: (1) participants who withdrew in the middle of a low-carbohydrate diet; (2) an experimental group or control group that had other surgical interventions or drug intervention as part of the research; (3) incomplete or incorrect data; (4) unavailable full-text literature; and (5) literature on nonrandomized controlled studies.(S3 Table)

### Outcome indicators

1. The main outcome indicators were the following: triglycerides, high-density lipoprotein cholesterol (HDL-C), total cholesterol (TC), low-density lipoprotein cholesterol (LDL-C), and body weight.The main observational indicators of each study are shown in S4 Table.
2. The secondary outcome indicators were fasting blood glucose and blood pressure.

### Data collection

Two authors (TTD and MG) independently screened the titles, abstracts, and full texts of the identified studies to determine their eligibility. Disagreements were resolved through consultation with a third author(GGS). The following data variables were recorded: first author’s name, year of publication, country of origin, study design (factorial, parallel, crossover, other), type of blinding (open, double blind), duration of follow-up, number of intervention groups, intervention regimen, total number of individuals and number of incident cases for each treatment group, mean age of each group, and risk estimates (mean) and corresponding 95% confidence intervals.

### Statistical analysis

All data processing and statistical analysis were performed using Review Manager (RevMan) software version 5.1 (http://www.ims.cochrane.org/revman/). We then stratified the duration of the low-carbohydrate diet intervention (i.e., less than 6 months, 6 to 11 months, 12 to 23 months, and 24 or more months). However, some outcome variables did not have enough data to allow an analysis across all four follow-up periods. Therefore, important individual indicators in the study were retained for analysis.The study were divided into an intervention group (a diet with less than 40% carbohydrates) and a control group(a diet with 45% to 55% carbohydrates).Heterogeneity among the studies was evaluated by calculating the I^2^ statistic and the chi^2^ test (significance level p<0.1),When I^2^ value is less than 50%, heterogeneity is small or nonexistent,whereas the I^2^ values of 50% or more indicate a substantial level of heterogeneity and values of 75% or more indicate considerable heterogeneity.We also performed a sensitivity analysis by removing each individual trial from the meta-analysis.

Some of the studies included in our meta-analysis differed in the units used to report lipid levels(mmol/L *vs* mg/dL). Therefore, we multiplied the values by 0.01129 to convert the triglycerides to mmol/L and by 0.02586 to convert the cholesterol to mmol/L.

## Results

### Literature search

**Fig 1:**
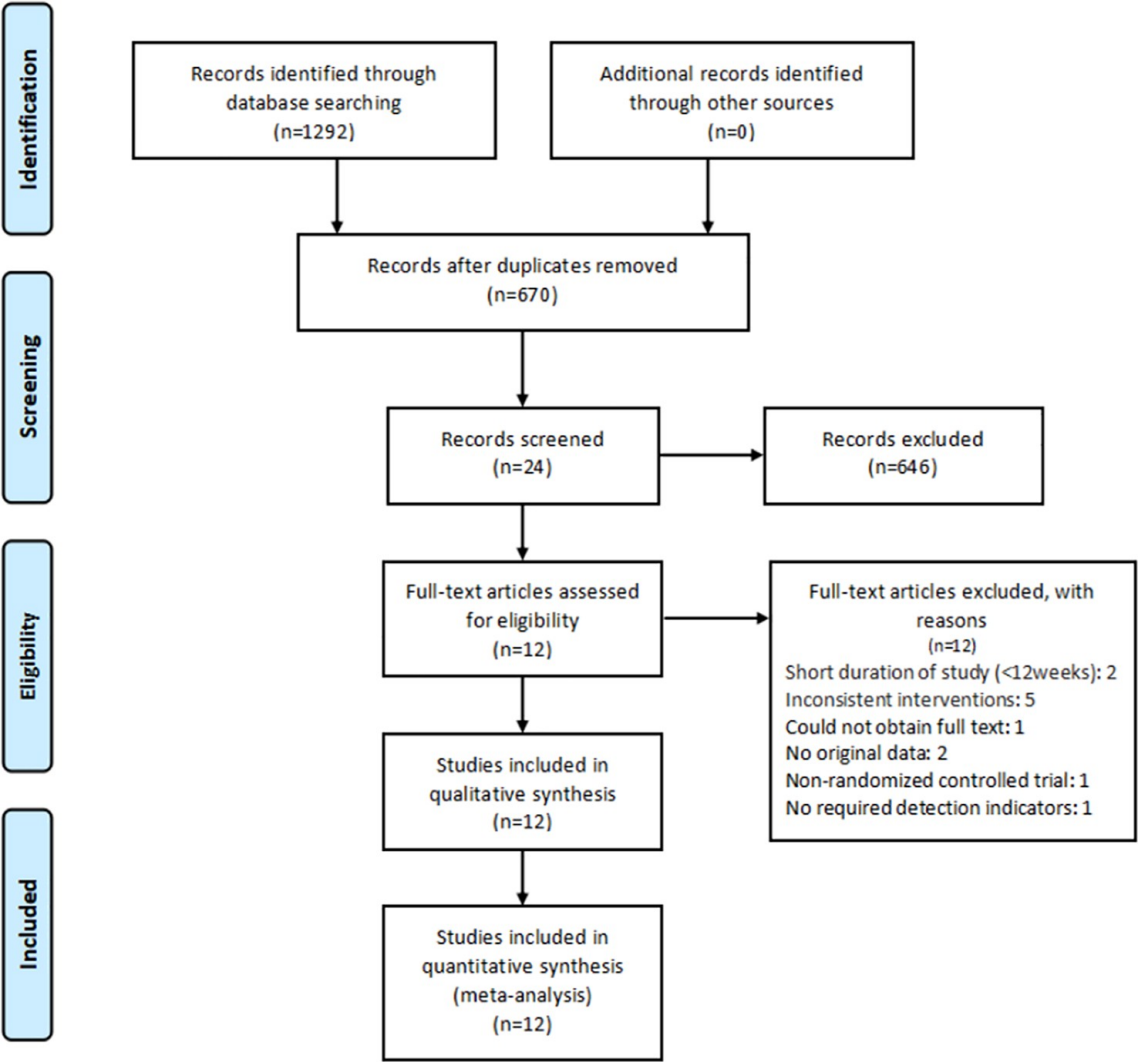
Flowchart of the meta-analysis.

A total of 1292 articles were identified during the literature search. After filtering for the inclusion and exclusion criteria, 24 studies were selected for full-text review. We excluded 12 full-text articles for the reasons given in S3 Table. Ultimately, 12 randomized controlled trials were included (Fig 1).

### Design characteristics

A total of 1640 patients were included in the 12 trials[15–26],including 820 in the observation group and 820 in the control group. The largest sample size was 403 cases, and the smallest was 42 cases. Of the twelve trials, five were conducted in the USA, three in Australia, two in the UK, one in Israel and one in China. The patients’ ages ranged from 31 to 65 years old. The intervention was a diet with less than 40% carbohydrates in the observation group and a diet with 45% to 55% carbohydrates in the control group. The baseline characteristics of the study participants and the study design characteristics are presented in S5 Table.

### Quality evaluation of the included studies

**Fig 2:**
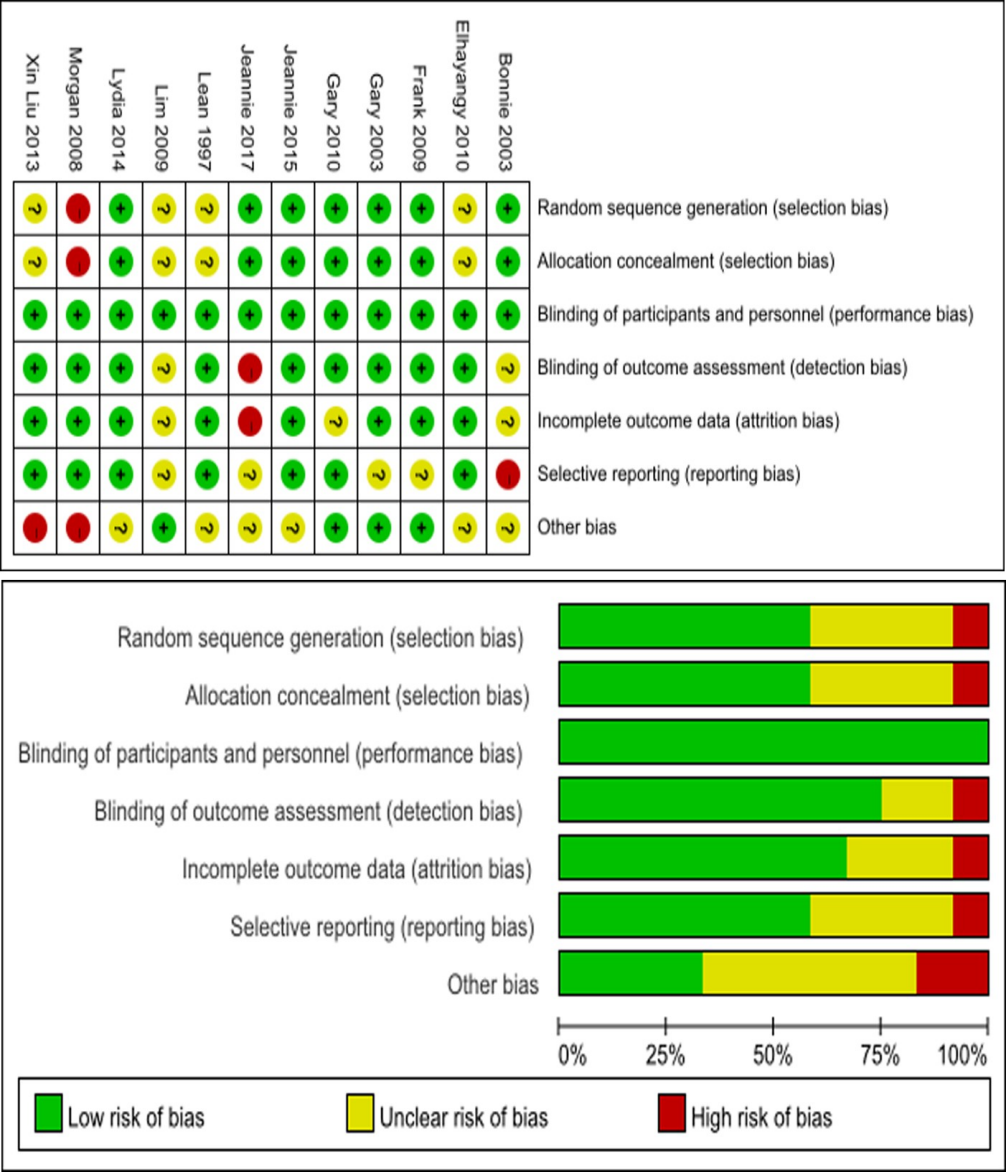
Cochrane risk assessment scale.

The quality of the included articles was assessed by the Cochrane risk assessment tool, which considers the following: (1) randomized method; (2) hidden distribution scheme; (3) blinding method; (4) loss of visit/withdrawal; (5) selective reporting; and (6) other sources of bias. Each study was assessed as having a ‘low risk of bias’ (A), an ‘unknown risk of bias’ (B), or a ‘high risk of bias’ (C) according to the probability of bias (Fig 2).

### Meta-analysis results

#### Main outcome indicators for cardiovascular risk factors: Triglycerides

**Fig 3:**
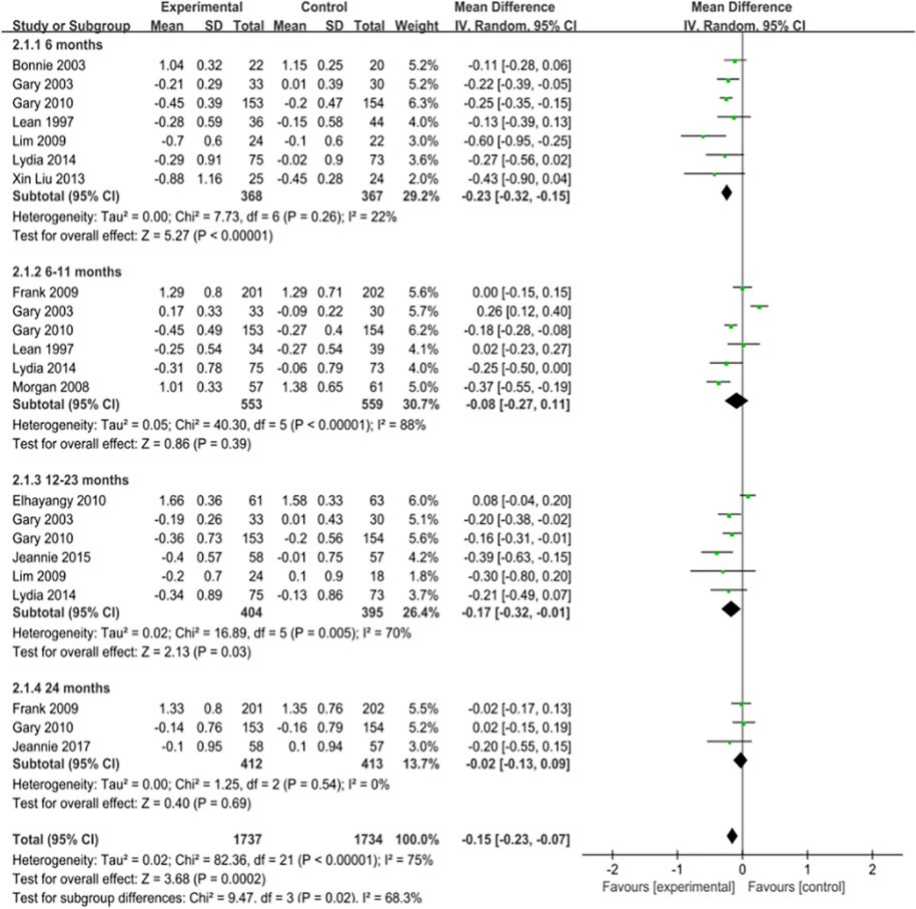
Forest plot for Triglycerides.

Compared with those of the control group, the triglyceride levels of the experimental group decreased by 0.15mmol/l (95% CI -0.23 to -0.07) (Fig 3); low-carbohydrate diet interventions lasting less than 6 months decreased these levels by 0.23mmol/l (95% CI -0.32 to -0.15), and those of 12–23 months decreased the levels by 0.17mmol/l (95% CI -0.32 to -0.01).As the meta-analysis showed I^2^ = 75%, P = 0.001 and there was a high degree of heterogeneity, a random-effects model was used for the combined analysis. Because of the heterogeneity, a subgroup analysis was conducted to investigate the source of heterogeneity. Meta-analysis was performed according to the subgroup of the region. Compared with the control group, the heterogeneity was reduced to I^2^ = 54%, P = 0.02, Next, a sensitivity analysis was performed by excluding each study one at a time. The results showed that when the study by Elhayany literature was excluded, the results remained unchanged,but the heterogeneity decreased significantly to -0.16mmol/l (95% CI -0.24 to -0.08), I^2^ = 39%, possibly because of the small number of samples in this study. (S6 Table, S1 File). The results of the publication bias analysis showed that the funnel plot was not symmetric (S1 Fig). The asymmetry of the funnel plot may have been caused by publication bias and other issues.

#### HDL-C

**Fig 4:**
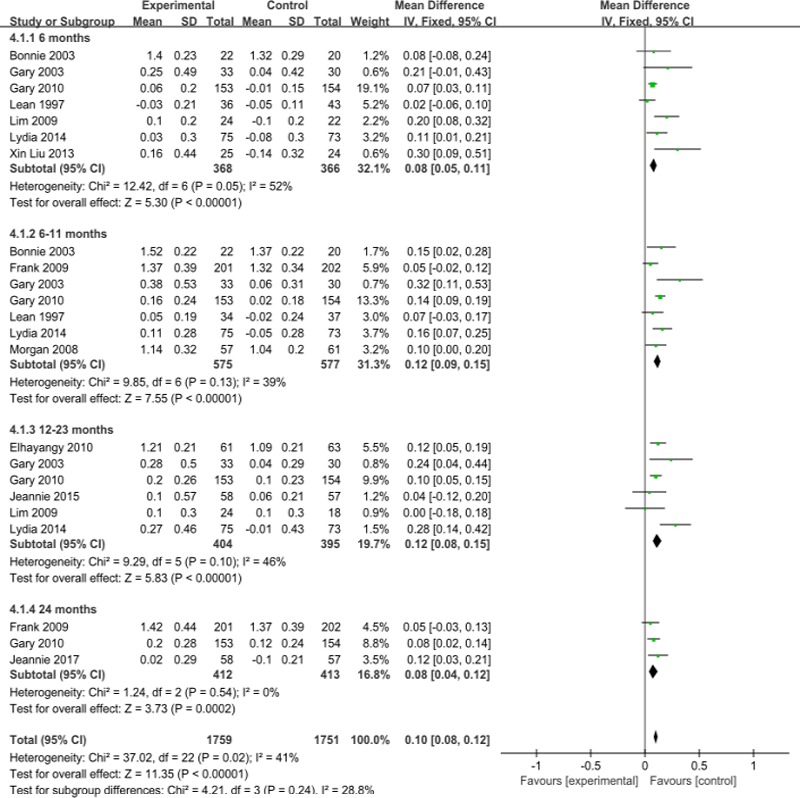
Forest plot for high-density lipoprotein.

Compared with that in the control group, the observation group showed an increased plasma HDL-C level, and the overall level increased by 0.1mmol/l (95%CI 0.08 to 0.12;) (Fig 4). The data were divided into different study times, and the increase in plasma HDL-C levels was 0.08mmol/l (95%CI 0.27 to 0.57) for interventions lasting less than 6 months,0.12mmol/l (95% CI 0.09 to 0.15) for those lasting 6–11 months,0.12mmol/l for those lasting 12–23 months (95%CI 0.08 to 0.15), and 0.08mmol/l for those lasting 24 months (95%CI 0.04 to 0.12). The meta-analysis showed I^2^ = 41%, P = 0.02, indicating moderate heterogeneity; in an analysis stratified by region, age, and sample size, the result was I^2^ = 0%, P = 0.52. (S7 Table) The increase in heterogeneity may have been due to the area, age, etc., as included in the study supplement. (S2 File) The publication bias analysis showed that the funnel plot was symmetric (S2 Fig).

#### Total cholesterol

**Fig 5:**
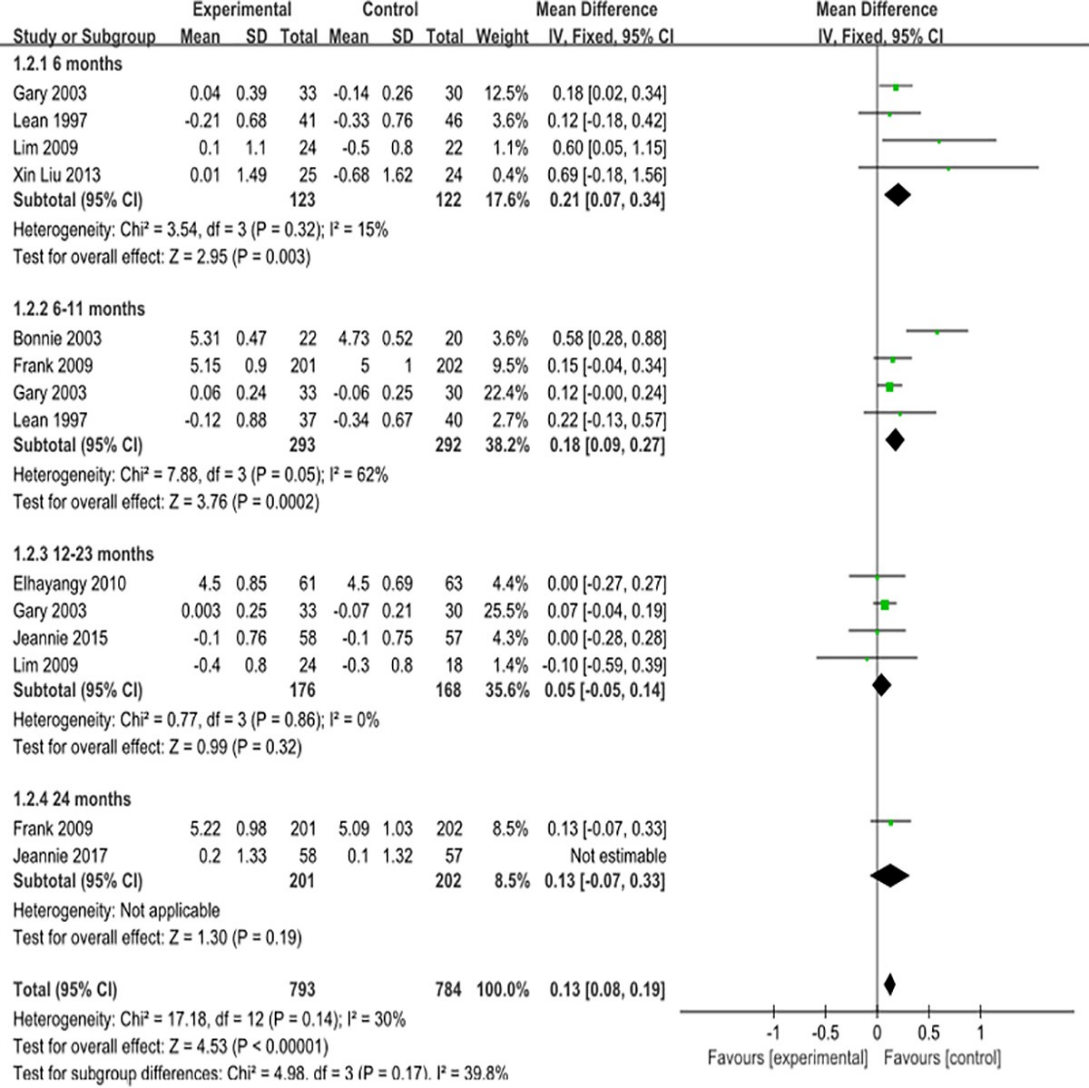
Forest plot for total cholesterol.

Regarding total cholesterol, a slight change was shown for the overall low-carbohydrate diet. However, there was no significant change in the data corresponding to low-carbohydrate diets lasting 12–23 months and over 24 months, which were 0.05mmol/l(95%CI -0.05 to 0.14) and 0.13mmol/l(95%CI -0.06 to 0.31), respectively. (Fig 5);The publication bias analysis showed that the funnel plot was symmetric (S3 Fig).

#### LDL-C

**Fig 6:**
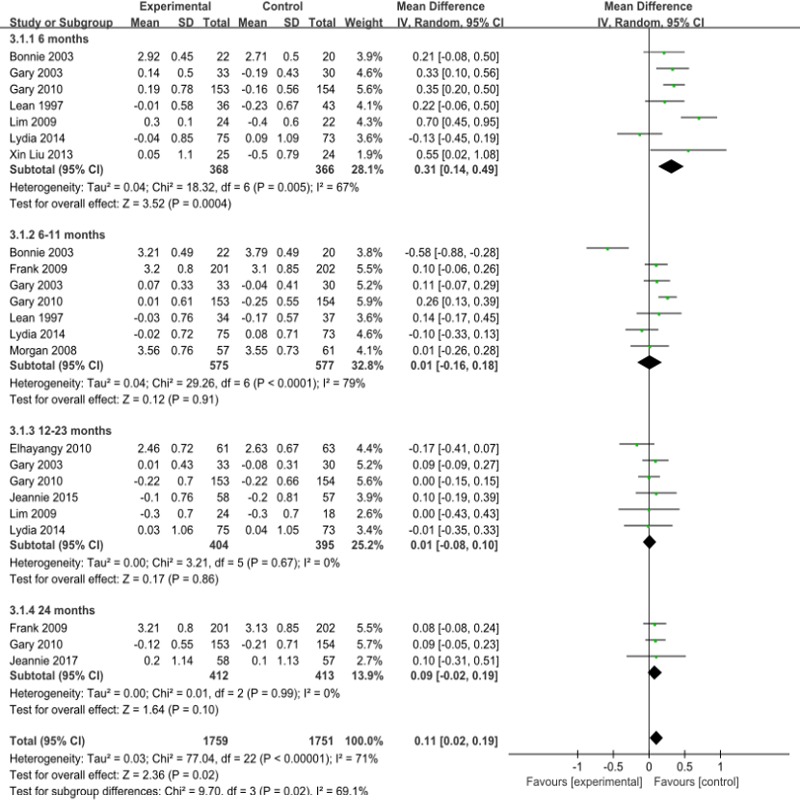
Forest plot for low density lipoprotein.

For plasma LDL-c, the overall low-carbohydrate diets correlation changed slightly (Fig 6).However,as the forest map shows, that there was no significant difference between the low-carbohydrate diet group and the control group at 6–11 months 0.01mmol/l(95%CI -0.16 to 0.10), 12–23 months 0.01mmol/l(95%CI -0.08 to 0.10), and 24 months (95%CI -0.02 to 0.19).The meta-analysis showed I^2^ = 71%, P = 0.0001, and there was a high degree of heterogeneity. The stratification analysis by region showed that I^2^ = 0%, P = 0.66.(S8 Table). Increases in heterogeneity may have been due to the circumstances of the areas included in the study. (S3 File). The publication bias analysis showed that the funnel plot was symmetric (S4 Fig).

#### Secondary outcome indicators for cardiovascular risk factors Weight

**Fig 7:**
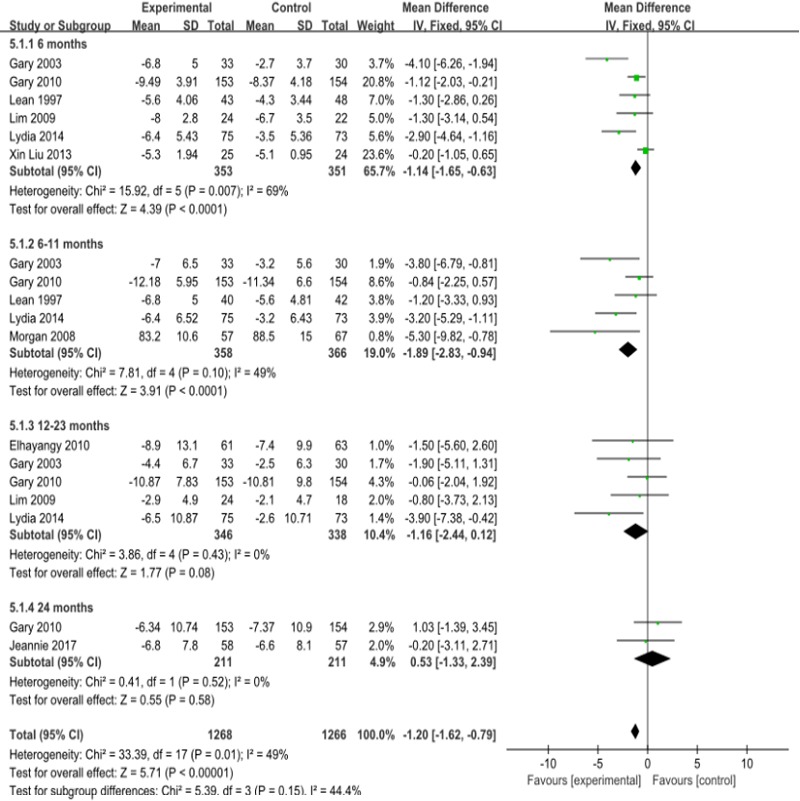
Forest plot for weight.

Compared with the subjects in the control group, those in the observation group lost weight (Fig 7); the change in weight was -1.58kg overall (95% CI -1.58 to -0.75),-1.14kg for interventions lasting less than 6 months of intervention (95%CI -1.65 to -0.63), and -1.73kg for interventions lasting 6–11 months (95% CI -2.7 to -0.76). The meta-analysis showed I^2^ = 49%, P = 0.01, and there was moderate heterogeneity. The stratification analysis by region showed that I^2^ = 17%, P = 0.29. (S9 Table). Increases in heterogeneity may have been due to the circumstances of the areas included in the study.(S4 File, S5 Fig)

#### Fasting plasma glucose

**Fig 8:**
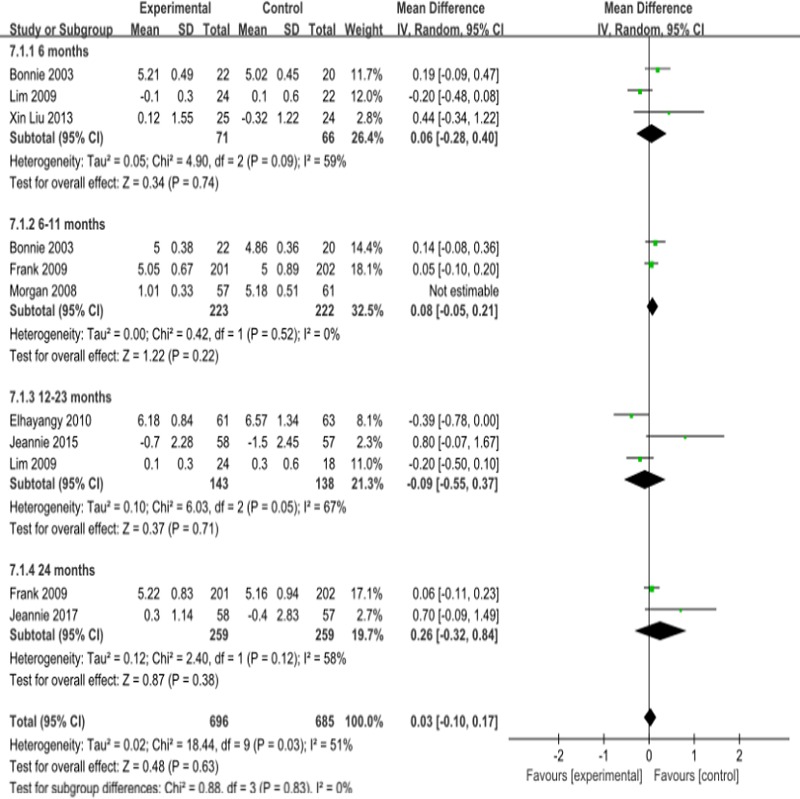
Forest plot for Fasting plasma glucose.

There was no significant change in the fasting blood glucose levels of the observation group compared with those of the control group 0.03mmol/l (95% CI -0.05 to 0.12,) (Fig 8).

#### Systolic blood pressure

**Fig 9:**
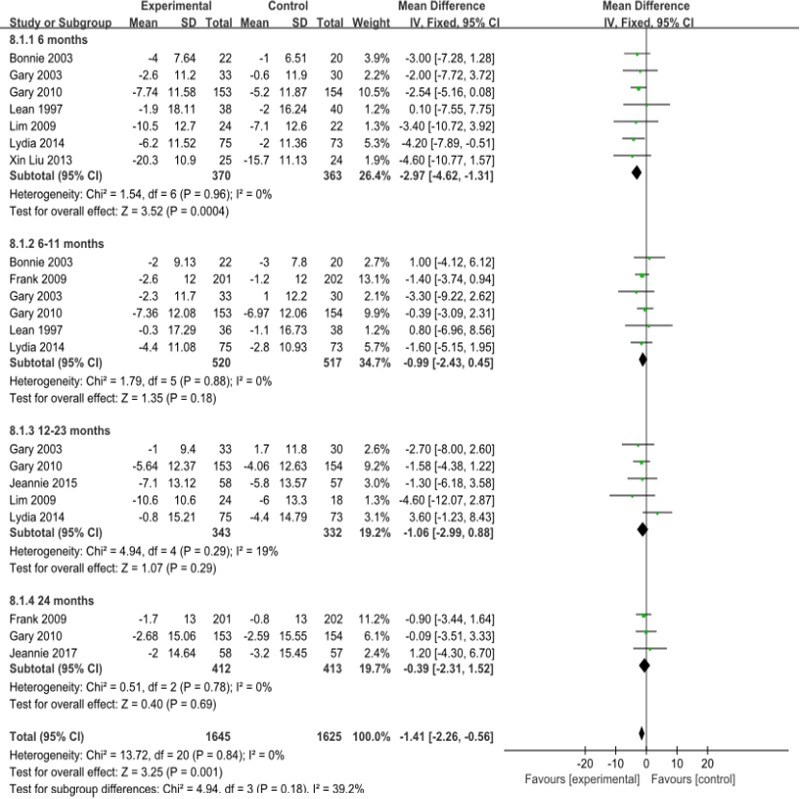
Forest plot for Systolic blood pressure.

Compared with that of the control group, the overall systolic blood pressure decreased as a whole by 1.41mmHg in the observation groups (95%CI- 2.26 to -0.56) (Fig 9), and it decreased significantly by 2.97mmHg in the group that received interventions lasting less than 6 months(95% CI -4.62 to—1.31).the meta-analysis showed that I^2^ = 0%, P = 0.84, without heterogeneity.The results of the publication bias analysis showed that the funnel plot was symmetric (S6 Fig).

#### Diastolic blood pressure

**Fig 10:**
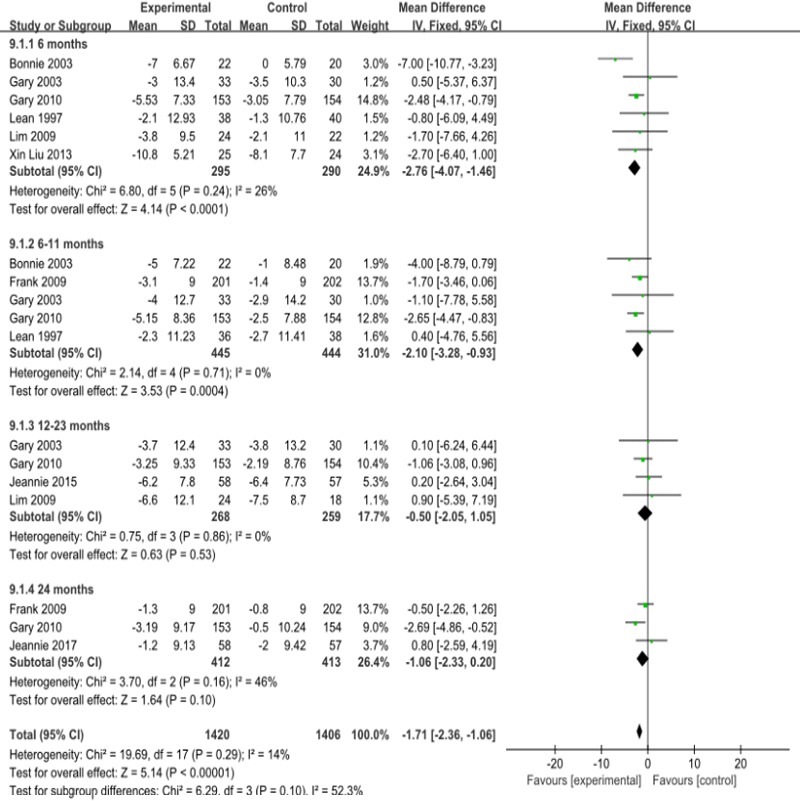
Forest plot for diastolic blood pressure.

Compared with that of the control group, the diastolic blood pressure of the observation groups decreased by 1.71mmHg overall (95% CI—2.36 to -1.06) (Fig 10), by 2.76mmHg in the group that received l less than 6 months of intervention (95% CI -4.07 to -1.46) and by 2.11mmHg in the group that received 6–11 months of intervention (95% CI—3.28 to -0.93). Meta analysis showed that I^2^ = 14%, P = 0.29, with low heterogeneity.The results of the publication bias analysis showed that the funnel plot was symmetric (Fig 10, S7 Fig).

**Sensitivity analysis**: After subtracting each of the included studies individually, the effect amount was combined and the results remained unchanged, Namely,triglyceride were -0.16mmol/l (95% CI -0.24 to -0.08), HDL was 0.1mmol/l (95% CI 0.08 to 0.12),TC was 0.13 mmol/l (95% CI 0.06 to 0.19), LDL was 0.10 mmol/l (0.01 to 0.19), body weight—was 1.1 kg (95% CI—1.53 to -0.68), fasting blood glucose was 0.02 mmol/l (95% CI—0.14 to 0.18), systolic blood pressure was—1.34 mm Hg (95% CI—2.21 to -0.48), and diastolic blood pressure was—1.71 mm Hg (95% CI—2.36 to -1.05).

## Discussion

This study conducted a meta-analysis aimed at studying the effects of low-carbohydrate diets on major cardiovascular risk factors. The overall effect of low-carbohydrate diets on cardiovascular risk factors, compared with the effects of a control diet, on cardiovascular risk factors tended to be favorable at less than 6 months and 6–11 months, but after 2 years of a low-carbohydrate diet, there was little effect on cardiovascular risk factors.

Although LDL-C showed a slight increase overall in the results of this meta-analysis, more favorable changes in several other lipid parameters (HDL-C and TG) were observed, and there were no significant changes in LDL-C after more than 6 months were observed. The results of several other meta-analyses were relatively consistent[27–30].A study by Lu et al[28] study showed an increase in LDL-C of 0.11 mmol/L (95%CI, 0.202 to 0.026) with the low-carbohydrate diets. The authors emphasized the beneficial HDL-C–raising effect of the low-carbohydrate diets of 0.066 mmol/L (95%CI, 0.10 to 0.033), equal to a 7.45% reduction in the relative risk of CVD.The low-carbohydrate diet appears more likely to improve cardiovascular risk factors.

Targeting LDL-C, inhibiting 3-hydroxy-3-methylglutaryl coenzymea (HMG-CoA) reductase activity with statins, reducing liver cholesterol production and up regulating the LDL receptor, are currently traditional prevention strategies for treating CVD and reducing mortality[31]; however, current clinical studies have found that this strategy reduces the CVD risk by less than 30%[32,33]. The main limitation of this strategy is the observed atherosclerotic complications of the participants. In a study by Teuta et al[30], LDL-C levels in nonfasting samples were quantified from low (<2.59 mmol/L [100 mg/dL]) to high (>4.14 mmol/L [160 mg/dL]) with these lipids,and risk was associated with quality parameters. This finding indicates that LDL-C-lowering treatment does not reduce the residual risk of cardiovascular events, indicating that even if the acceptable LDL-C target is met, there are risk factors other than LDL-C. The research data show that as the carbohydrates are limited in a low-carbohydrate diet, the increases in protein and fat intake will increase the concentration of plasma LDL-C; however, this elevation increases the LDL particle size from small to large, and atherosclerosis is caused by smaller LDL particles [34].That is, cholesterol-rich large buoyancy low-density lipoprotein particles (lbLDL) have been shown to have lower atherogenic atherosclerosis potential, while small density particles (sdLDLs) and moderate concentrations of low-density lipoprotein particles are more strongly correlated with cardiovascular disease outcomes[35,36], sdLDL particles (phenotype B) were more strongly associated with CVD outcomes than lbLDL particles (phenotype A). After a low-carbohydrate diet, the risk of CVD is reduced, while the opposite occurs after a high-carbohydrate diet[37].

The INTERHEART study showed that people with abnormal blood lipid levels are 3 times more likely to have cardiovascular disease than those with normal blood lipid levels [38]. Although patients with hyperlipidemia can control their blood lipid levels with drugs, more than 50% of patients cannot receive drug treatment due to side effects, financial constraints or other reasons [39]. The results showed that triglyceride levels decreased with a low-carbohydrate diet intervention of less than 6 months and an intervention period of 11–23 months -0.23 mmol/l (95%CI -0.32 to -0.15); and—0.17 mmol/l (95%CI—0.32 to -0.01), respectively. In the study and analysis, HDL-C increased in four studies under the low-carbohydrate diet intervention. An increase in HDL-C in a low-carbohydrate diet may be associated with an increase in dietary fat intake [40]. Triglycerides are a key indicator for measuring the risk of cardiovascular diseases,and a reduction in triglycerides can protect the cardiovascular system and reduce the risk of cardiovascular diseases [41,42].Rader DJ et al[43]. showed that elevated HDL cholesterol levels can reduce certain cardiovascular risk factors.However, the mechanism underlying the significant increase in the HDL-C level of subjects under going the low-carbohydrate diet intervention is still not clear, and more research on the underlying mechanism is needed. Of clinical significance, is the finding that the increase in the HDL-C level was generally considered beneficial.

Of the 12 original randomized controlled trials included in our meta-analysis, 11 analyzed body weight,which decreased significantly in the observation groups compared with the control group -1.58 kg (95% CI -1.58 to -0.75). Among the observation groups, subjects who underwent less than 6 months of intervention had a weight change of -1.14kg(95%CI -1.65 to -0.63) and and those whose intervention lasted 6–11 months had a weight change of -1.73kg(95%CI -2.7 to -0.76),showing the most obvious decreases in body weight. These results support recent weight-loss recommendations because most calorie-reducing diets lead to clinically important weight loss as long as the diet is maintained [11,44–47]. After remaining on a low-carbohydrate diet for more than 1 year, the observation group showed was no significant difference from the control group, indicating that the low-carbohydrate diet has certain benefits for weight loss in the short term and no significant impact in the long term, possibly due to the poor compliance by the subjects. For multiple reasons, a large amount of research data is needed to demonstrate the long-term effects of a low-carbohydrate diet on body weight. The overall results obtained in this study favored a low-carbohydrate diet, and although the magnitude of the changes was small, there were still changes that were associated with low-carbohydrate diets. The overall change in weight loss was associated with favorable changes in cardiovascular risk factors.

Although the RCTs included in this study were mostly high-quality studies, there were also deficiencies in this study: (1) There are still some potential confounding factors in this study. For example, different dietary habits and preferences in different regions may increase the risk of selection bias. (2) The carbohydrate limits for the low-carbohydrate diets were not identical in each study, leading to clinical heterogeneity among the studies. (3) Most of the study interventions had short time courses and included less than 2 years of research. The results reflect the impact of only short-term low-carbohydrate diets on cardiovascular factors, and additional large-scale multicenter long-term clinical RCTs are needed to verify the results. (4)Finally, there may be other influencing factors that were not examined in the in subgroup analyses, such as the history of diabetes.

Overall, 12 reports were identified that met the predetermined criteria. This meta-analysis showed that a low-carbohydrate diet was significantly related to reductions in body weight, diastolic blood pressure, plasma triglycerides, and fasting glucose levels and an increase in HDL-C levels. The long-term effects of low-carbohydrate diets and their effects on clinical end points (e.g., myocardial infarction, stroke, and total mortality) remain largely unknown and should be the subject of future studies.

In conclusion, the overall effect of a low-carbohydrate diet on cardiovascular risk factors tended to be favorable at less than 6 months and 6–11 months, but after 2 years of a low-carbohydrate diet, there was no significant effect on cardiovascular risk factors.

## Supporting information

S1 File Subgroup analysis of forest plot for triglyceride. (ZIP)

S2 File Subgroup analysis of forest plot for HDL. (ZIP)

S3 File Subgroup analysis of forest plot for LDL. (ZIP)

S4 File Subgroup analysis of forest plot for weight. (ZIP)

S1 Fig Funnel plot for triglycerides. (TIF)

S2 Fig Funnel plot for high-density lipoprotein. (TIF)

S3 Fig Funnel plot for total cholesterol. (TIF)

S4 Fig Funnel plot for low density lipoprotein. (TIF)

S5 Fig Funnel plot for weight. (TIF)

S6 Fig Funnel plot for systolic blood pressre. (TIF)

S7 Fig Funnel plot for diastolic blood pressure. (TIF)

S1 Table PRISMA checklist. (DOC)

S2 Table Search strategy in PubMed and embase. (DOCX)

S3 Table Excluded studies and reasons for exclusion. (DOCX)

S4 Table The main indicators observed in each study. (DOCX)

S5 Table General characteristics of included literature. (DOCX)

S6 Table Subgroup analysis of major cardiovascular risk factors triglyceride. (DOCX)

S7 Table Subgroup analysis of major cardiovascular risk factors HDL. (DOCX)

S8 Table Subgroup analysis of major cardiovascular risk factors LDL. (DOCX)

S9 Table Subgroup analysis of major cardiovascular risk factors weight. (DOCX)
