# Supplementary figures and images for: The effects of low-carbohydrate diets on cardiovascular risk factors: A meta-analysis
Source: PLoS One. 2020 Jan 14;15(1):e0225348. doi: 10.1371/journal.pone.0225348 (PMC6959586; doi:10.1371/journal.pone.0225348)

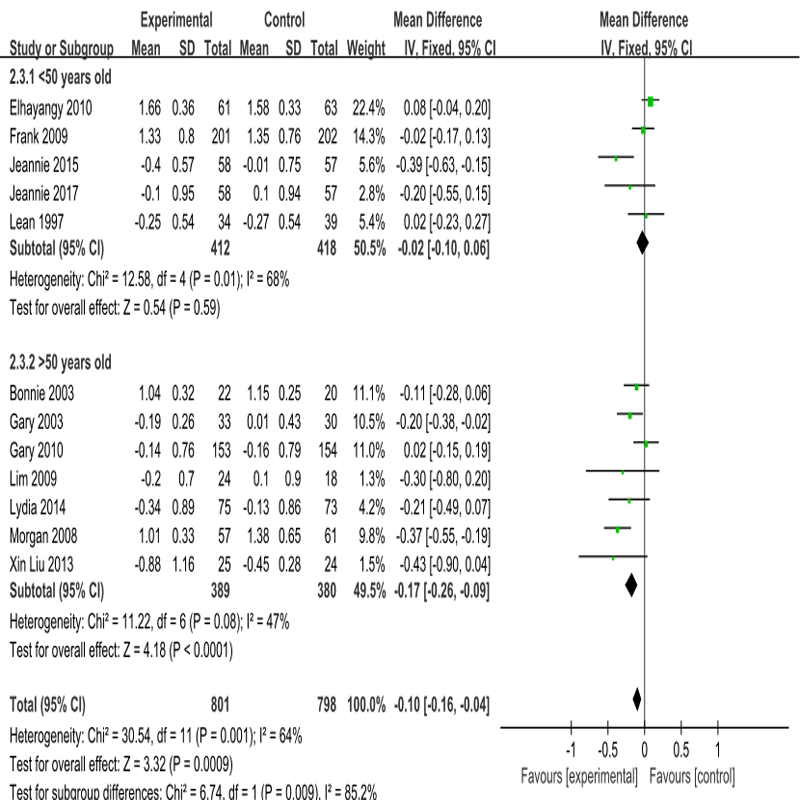

Supplement: S1 File — (ZIP) [file pone.0225348.s001.zip › S1 File/TAG-age.tif]

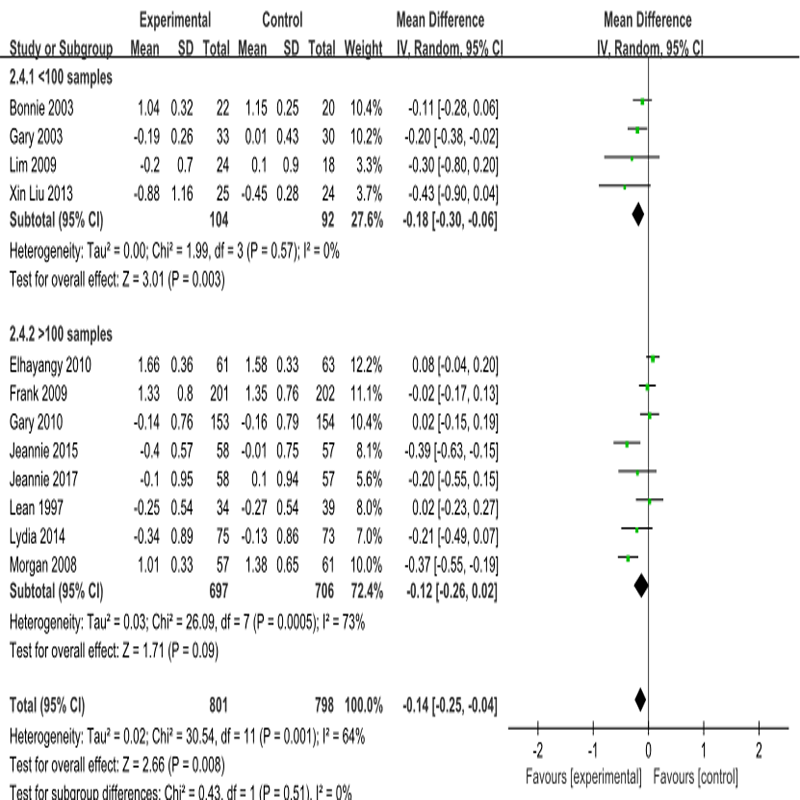

Supplement: S1 File — (ZIP) [file pone.0225348.s001.zip › S1 File/TAG-samples.tif]

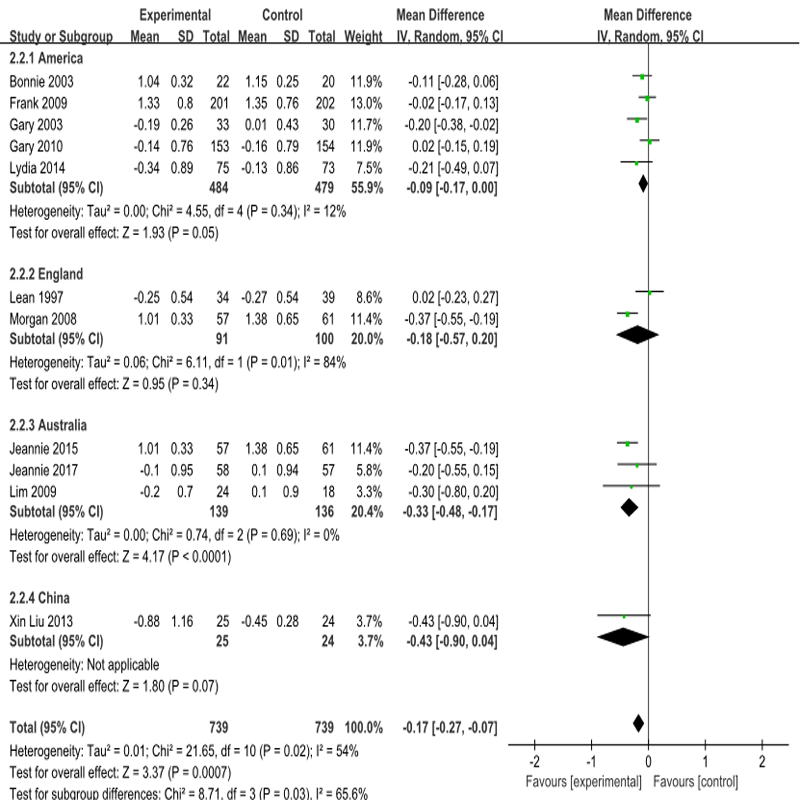

Supplement: S1 File — (ZIP) [file pone.0225348.s001.zip › S1 File/TAG-state.tif]

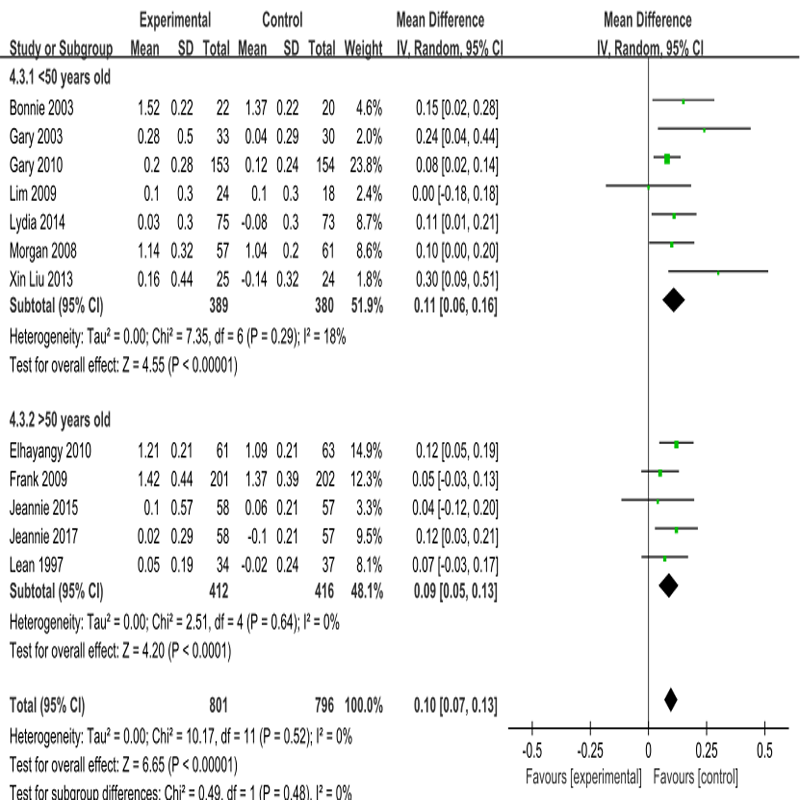

Supplement: S2 File — (ZIP) [file pone.0225348.s002.zip › S2 File/HDL-age.tif]

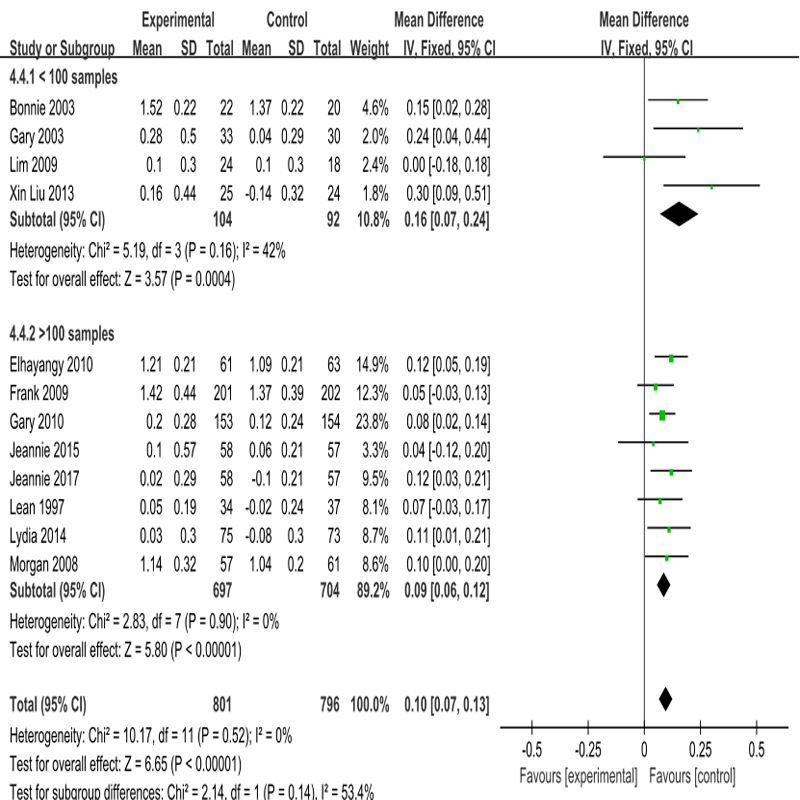

Supplement: S2 File — (ZIP) [file pone.0225348.s002.zip › S2 File/HDL-samples.tif]

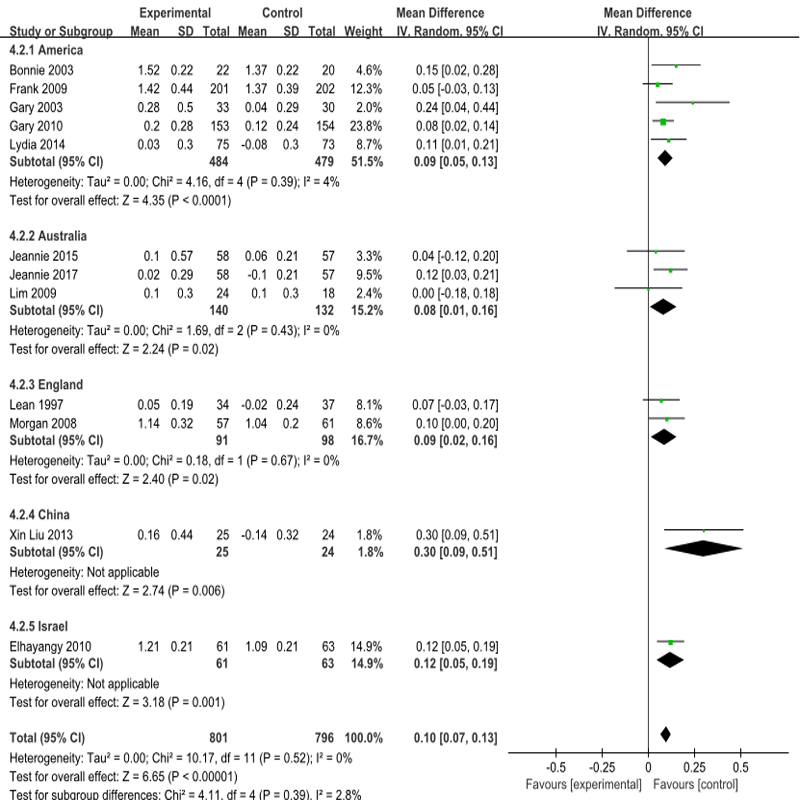

Supplement: S2 File — (ZIP) [file pone.0225348.s002.zip › S2 File/HDL-state.tif]

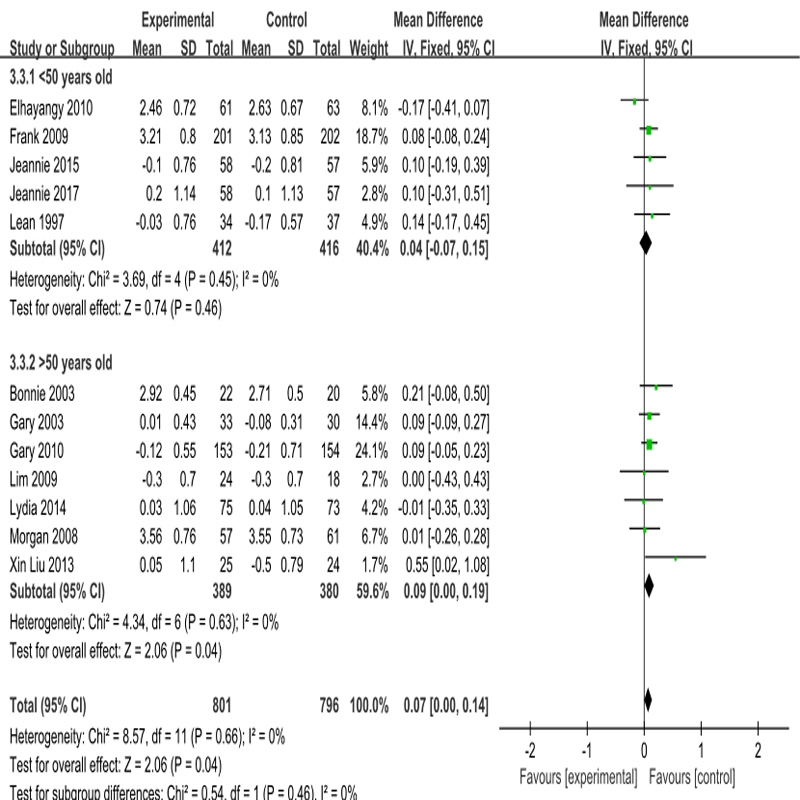

Supplement: S3 File — (ZIP) [file pone.0225348.s003.zip › S3 File/LDL-age.tif]

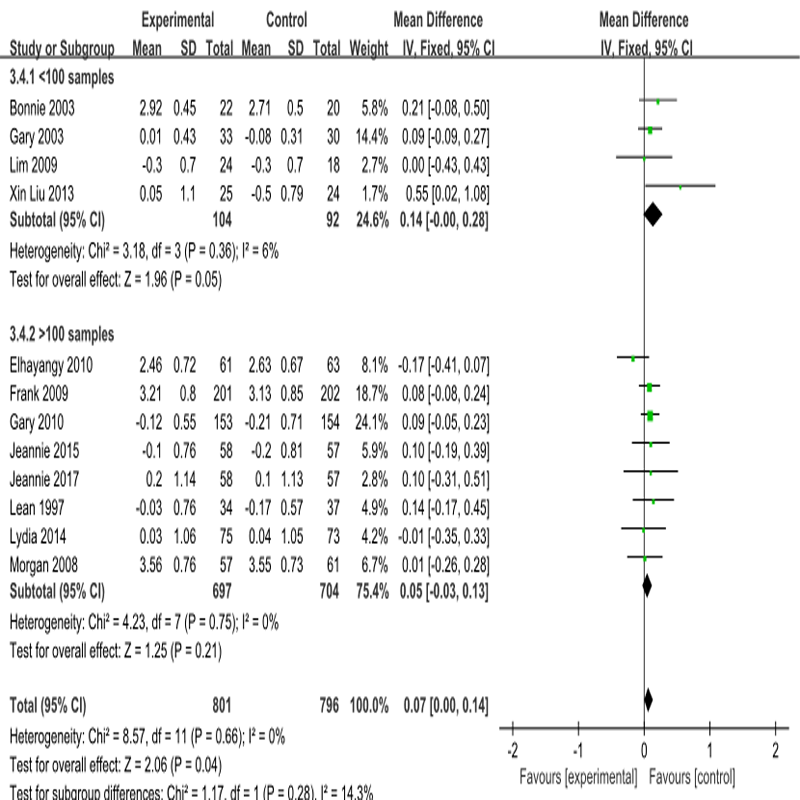

Supplement: S3 File — (ZIP) [file pone.0225348.s003.zip › S3 File/LDL-samples.tif]

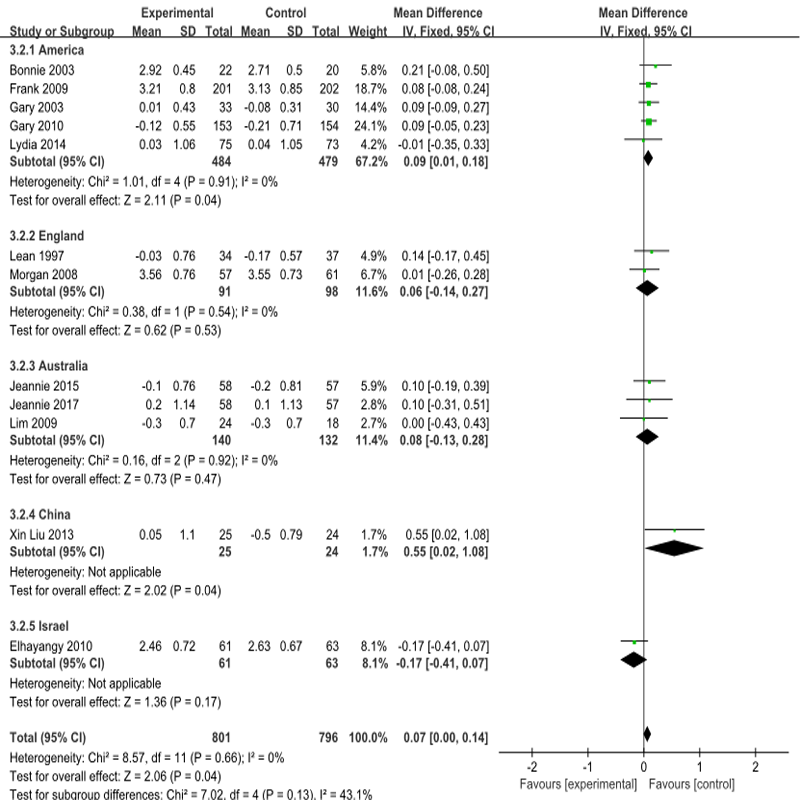

Supplement: S3 File — (ZIP) [file pone.0225348.s003.zip › S3 File/LDL-state.tif]

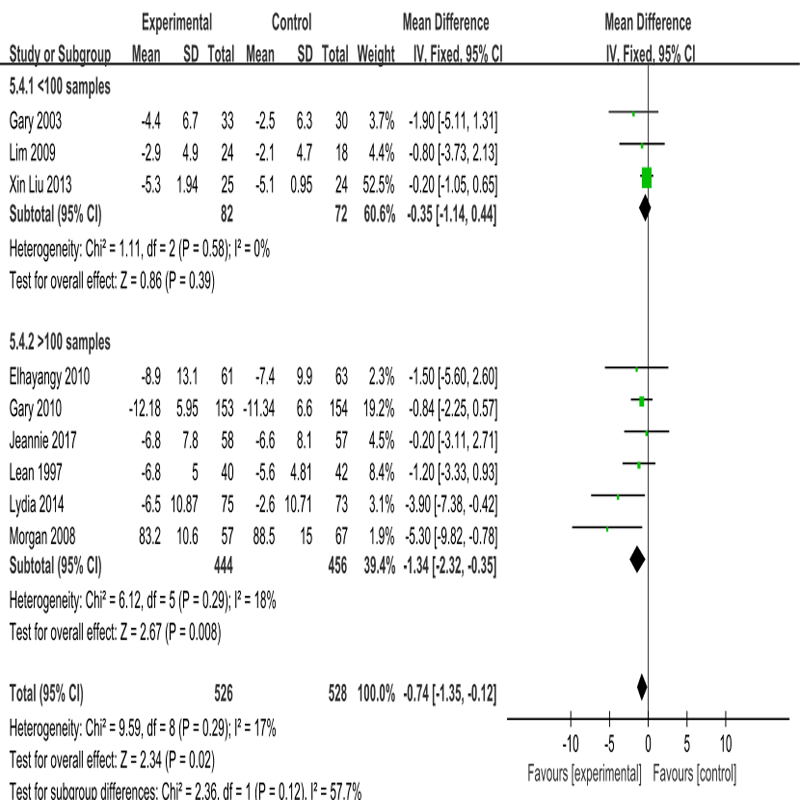

Supplement: S4 File — (ZIP) [file pone.0225348.s004.zip › S4 File/weight-samples.tif]

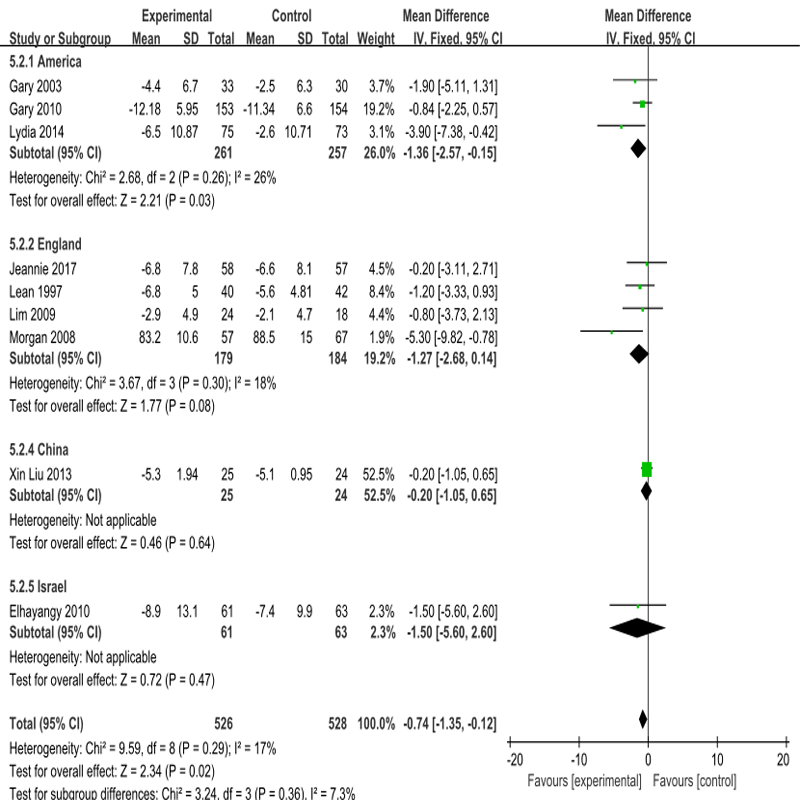

Supplement: S4 File — (ZIP) [file pone.0225348.s004.zip › S4 File/weight-state.tif]

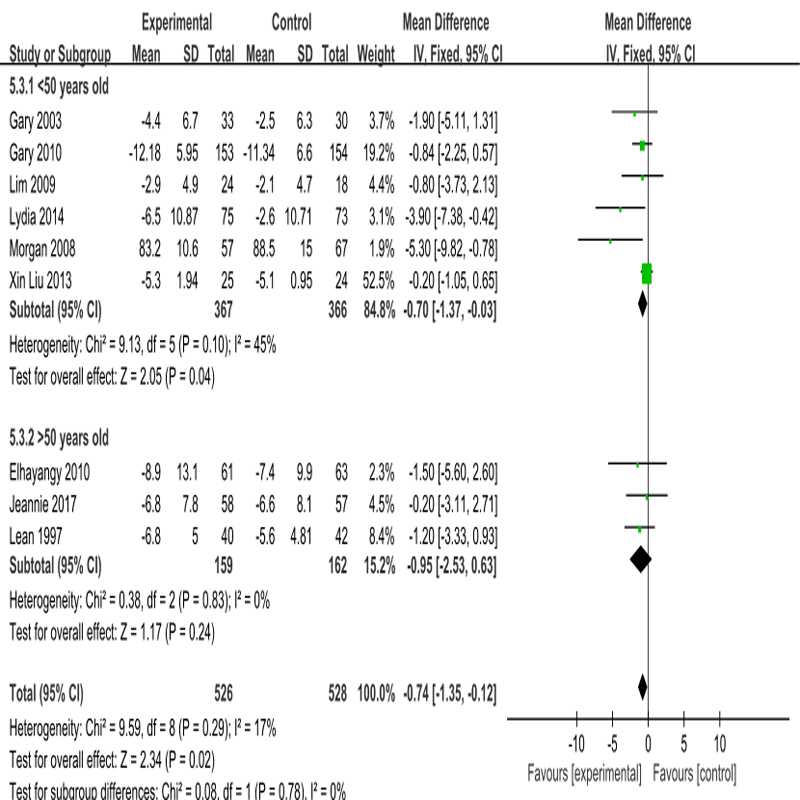

Supplement: S4 File — (ZIP) [file pone.0225348.s004.zip › S4 File/weihgt-age.tif]

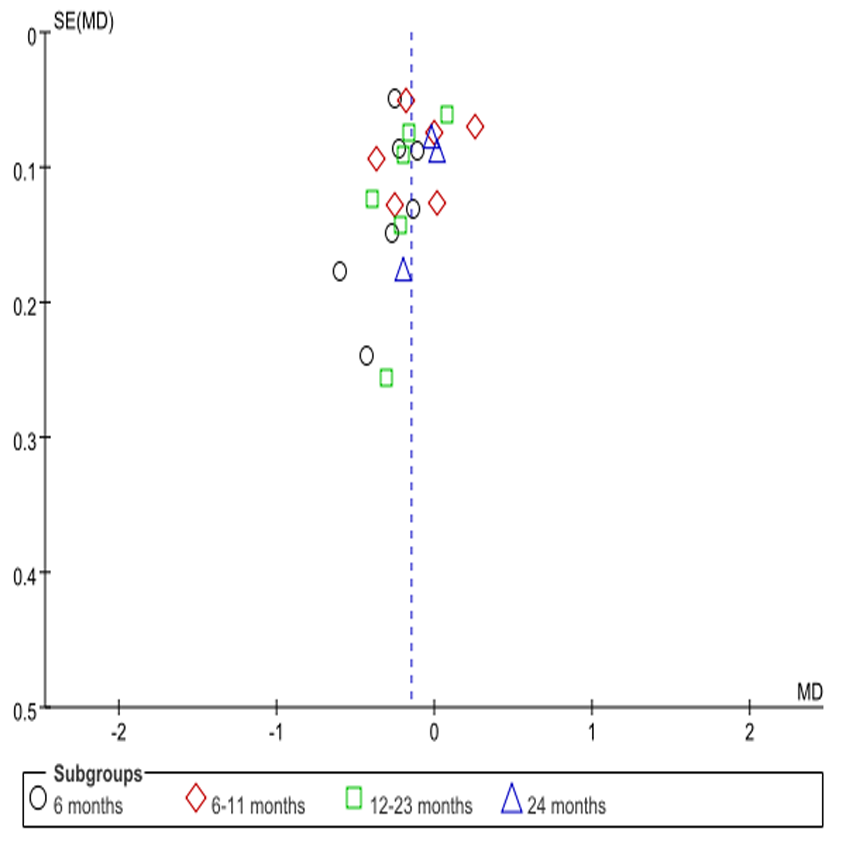

Supplement: S1 Fig — (TIF) [file pone.0225348.s005.tif]

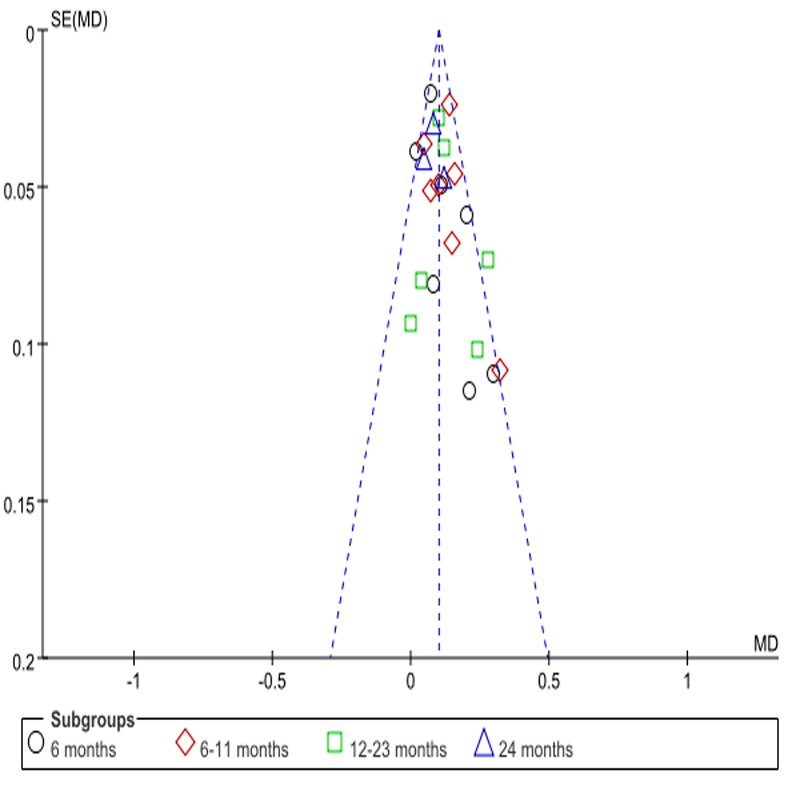

Supplement: S2 Fig — (TIF) [file pone.0225348.s006.tif]

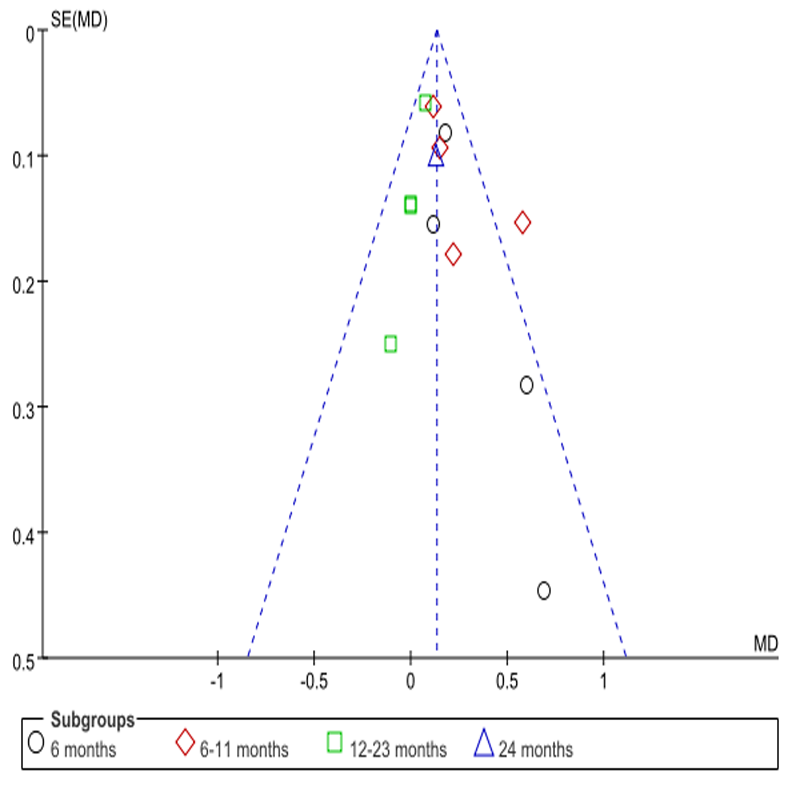

Supplement: S3 Fig — (TIF) [file pone.0225348.s007.tif]

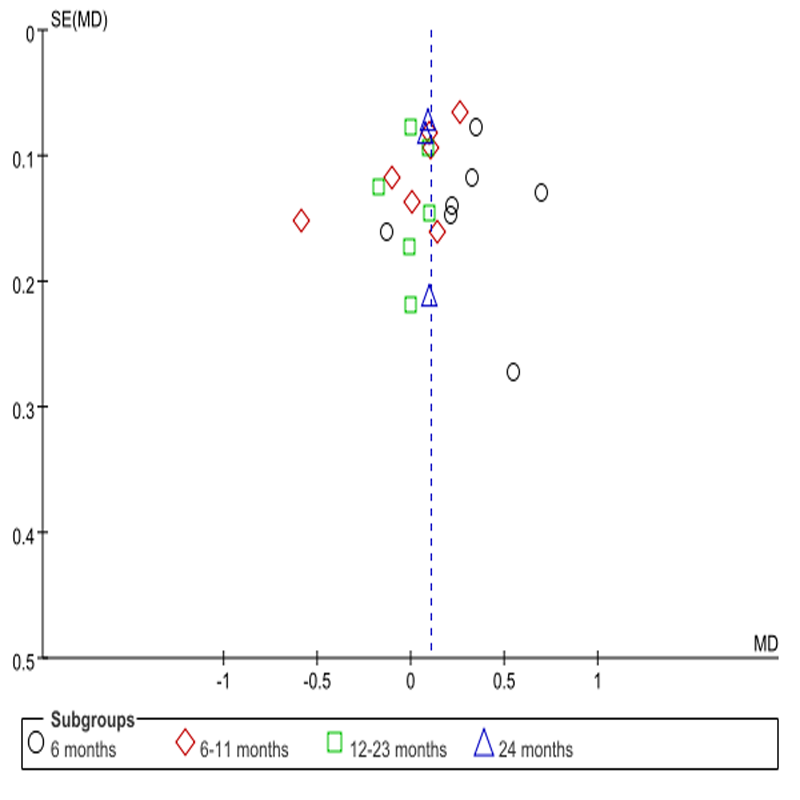

Supplement: S4 Fig — (TIF) [file pone.0225348.s008.tif]

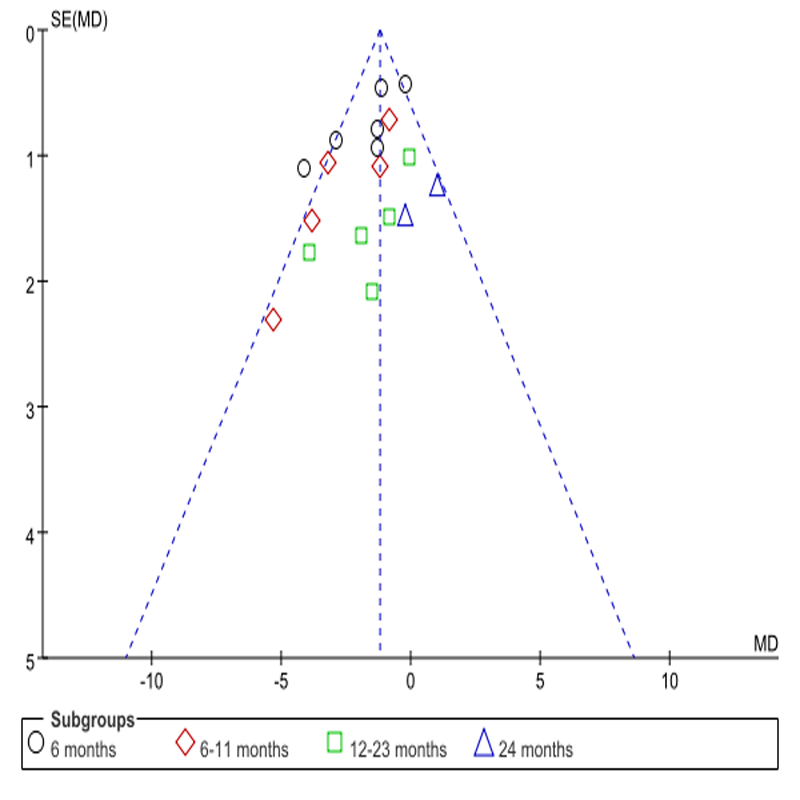

Supplement: S5 Fig — (TIF) [file pone.0225348.s009.tif]

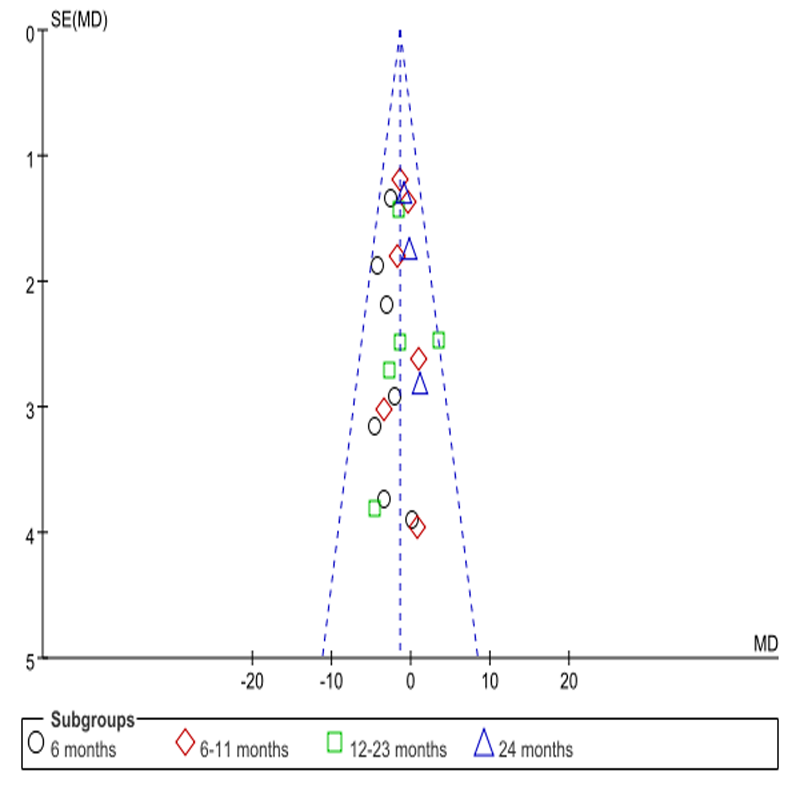

Supplement: S6 Fig — (TIF) [file pone.0225348.s010.tif]

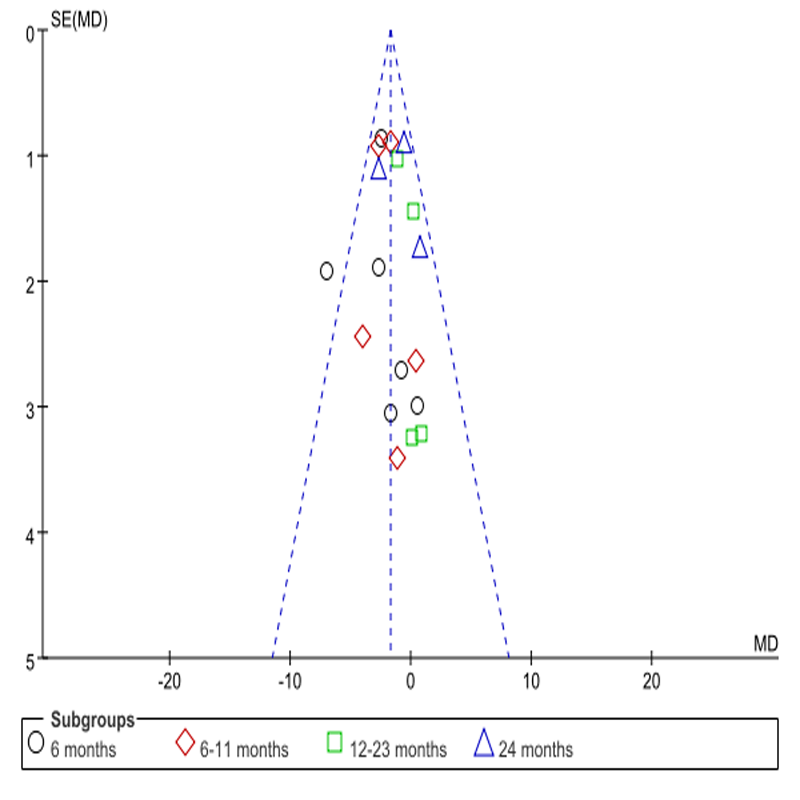

Supplement: S7 Fig — (TIF) [file pone.0225348.s011.tif]
