# Supplementary material for: The effects of low-carbohydrate diets on cardiovascular risk factors: A meta-analysis
Source: PLoS One. 2020 Jan 14;15(1):e0225348. doi: 10.1371/journal.pone.0225348 (PMC6959586; doi:10.1371/journal.pone.0225348)
Supplement: S2 Table — (DOCX) [file pone.0225348.s013.docx]

**S1 Table Search strategy in PubMed and Embase**

| **Table S1 Search strategy** |
| --- |
| **Ovid PubMed** |
| \| #10 \| Search ((((("Diet, Carbohydrate-Restricted"[Mesh]) OR ((((((((((((((((((Diet, Carbohydrate Restricted[Title/Abstract]) OR Diet, Low Carbohydrate[Title/Abstract]) OR Carbohydrate Diet, Low[Title/Abstract]) OR Carbohydrate Diets, Low[Title/Abstract]) OR Diets, Low Carbohydrate[Title/Abstract]) OR Low Carbohydrate Diets[Title/Abstract]) OR Carbohydrate-Restricted Diet[Title/Abstract]) OR Carbohydrate Restricted Diet[Title/Abstract]) OR Carbohydrate-Restricted Diets[Title/Abstract]) OR Diets, Carbohydrate-Restricted[Title/Abstract]) OR Low-Carbohydrate Diet[Title/Abstract]) OR Diet, Low-Carbohydrate[Title/Abstract]) OR Diets, Low-Carbohydrate[Title/Abstract]) OR Low Carbohydrate Diet[Title/Abstract]) OR Low-Carbohydrate Diets[Title/Abstract]) OR low-carbohydrate diet[Title/Abstract]) OR Atkins Diet[Title/Abstract]) OR Diet, Atkins[Title/Abstract]))) AND (("Cardiovascular Diseases"[Mesh]) OR (((Cardiovascular Disease[Title/Abstract]) OR Disease, Cardiovascular[Title/Abstract]) OR Diseases, Cardiovascular[Title/Abstract])))) AND ((((randomized controlled trial) OR controlled clinical trial) OR randomly) OR trials) Sort by: Best Match \| \| --- \| --- \| \| #9 \| Search (((randomized controlled trial) OR controlled clinical trial) OR randomly) OR trials Sort by: Best Match \| \| #8 \| Search ((("Diet, Carbohydrate-Restricted"[Mesh]) OR ((((((((((((((((((Diet, Carbohydrate Restricted[Title/Abstract]) OR Diet, Low Carbohydrate[Title/Abstract]) OR Carbohydrate Diet, Low[Title/Abstract]) OR Carbohydrate Diets, Low[Title/Abstract]) OR Diets, Low Carbohydrate[Title/Abstract]) OR Low Carbohydrate Diets[Title/Abstract]) OR Carbohydrate-Restricted Diet[Title/Abstract]) OR Carbohydrate Restricted Diet[Title/Abstract]) OR Carbohydrate-Restricted Diets[Title/Abstract]) OR Diets, Carbohydrate-Restricted[Title/Abstract]) OR Low-Carbohydrate Diet[Title/Abstract]) OR Diet, Low-Carbohydrate[Title/Abstract]) OR Diets, Low-Carbohydrate[Title/Abstract]) OR Low Carbohydrate Diet[Title/Abstract]) OR Low-Carbohydrate Diets[Title/Abstract]) OR low-carbohydrate diet[Title/Abstract]) OR Atkins Diet[Title/Abstract]) OR Diet, Atkins[Title/Abstract]))) AND (("Cardiovascular Diseases"[Mesh]) OR (((Cardiovascular Disease[Title/Abstract]) OR Disease, Cardiovascular[Title/Abstract]) OR Diseases, Cardiovascular[Title/Abstract])) Sort by: Best Match \| \| #7 \| Search ("Cardiovascular Diseases"[Mesh]) OR (((Cardiovascular Disease[Title/Abstract]) OR Disease, Cardiovascular[Title/Abstract]) OR Diseases, Cardiovascular[Title/Abstract]) Sort by: Best Match \| \| #6 \| Search ((Cardiovascular Disease[Title/Abstract]) OR Disease, Cardiovascular[Title/Abstract]) OR Diseases, Cardiovascular[Title/Abstract] Sort by: Best Match \| \| #5 \| Search "Cardiovascular Diseases"[Mesh] Sort by: Best Match \| \| #4 \| Search ("Diet, Carbohydrate-Restricted"[Mesh]) OR ((((((((((((((((((Diet, Carbohydrate Restricted[Title/Abstract]) OR Diet, Low Carbohydrate[Title/Abstract]) OR Carbohydrate Diet, Low[Title/Abstract]) OR Carbohydrate Diets, Low[Title/Abstract]) OR Diets, Low Carbohydrate[Title/Abstract]) OR Low Carbohydrate Diets[Title/Abstract]) OR Carbohydrate-Restricted Diet[Title/Abstract]) OR Carbohydrate Restricted Diet[Title/Abstract]) OR Carbohydrate-Restricted Diets[Title/Abstract]) OR Diets, Carbohydrate-Restricted[Title/Abstract]) OR Low-Carbohydrate Diet[Title/Abstract]) OR Diet, Low-Carbohydrate[Title/Abstract]) OR Diets, Low-Carbohydrate[Title/Abstract]) OR Low Carbohydrate Diet[Title/Abstract]) OR Low-Carbohydrate Diets[Title/Abstract]) OR low-carbohydrate diet[Title/Abstract]) OR Atkins Diet[Title/Abstract]) OR Diet, Atkins[Title/Abstract])Sort by: Best Match \| \| #3 \| Search (((((((((((((((((Diet, Carbohydrate Restricted[Title/Abstract]) OR Diet, Low Carbohydrate[Title/Abstract]) OR Carbohydrate Diet, Low[Title/Abstract]) OR Carbohydrate Diets, Low[Title/Abstract]) OR Diets, Low Carbohydrate[Title/Abstract]) OR Low Carbohydrate Diets[Title/Abstract]) OR Carbohydrate-Restricted Diet[Title/Abstract]) OR Carbohydrate Restricted Diet[Title/Abstract]) OR Carbohydrate-Restricted Diets[Title/Abstract]) OR Diets, Carbohydrate-Restricted[Title/Abstract]) OR Low-Carbohydrate Diet[Title/Abstract]) OR Diet, Low-Carbohydrate[Title/Abstract]) OR Diets, Low-Carbohydrate[Title/Abstract]) OR Low Carbohydrate Diet[Title/Abstract]) OR Low-Carbohydrate Diets[Title/Abstract]) OR low-carbohydrate diet[Title/Abstract]) OR Atkins Diet[Title/Abstract]) OR Diet, Atkins[Title/Abstract]Sort by: Best Match \| \| #2 \| Search ((((Diet, Carbohydrate Restricted) AND Diet, Low Carbohydrate) AND Carbohydrate Diet, Low) AND Carbohydrate Diets, Low) AND Diets, Low Carbohydrate Sort by: Best Match \| \| #1 \| Search "Diet, Carbohydrate-Restricted"[Mesh] Sort by: Best Match \| |

Embase

#25. #18 AND #22 AND #23

#24. 'randomized controlled trial'/exp

#23. 'randomized controlled trial'/exp

#22. #19 OR #20 OR #21

#21. 'diseases, cardiovascular':ab,ti

#20. 'disease, cardiovascular':ab,ti

#19. 'cardiovascular disease'/exp

#18. #1 OR #2 OR #3 OR #4 OR #5 OR #6 OR #7 OR #8 OR

#9 OR #10 OR #11 OR #12 OR #13 OR #14 OR #15 OR

#16 OR #17

#17. 'diet, atkins':ab,ti

#16. 'atkins diet':ab,ti

#15. 'low-carbohydrate diets':ab,ti

#14. 'diets, low-carbohydrate':ab,ti

#13. 'diet, low-carbohydrate':ab,ti

#12. 'low-carbohydrate diet':ab,ti

#11. 'diets, carbohydrate-restricted':ab,ti

#10. 'carbohydrate-restricted diets':ab,ti

#9. 'carbohydrate restricted diet':ab,ti

#8. 'carbohydrate-restricted diet':ab,ti

#7. 'low carbohydrate diets':ab,ti

#6. 'diets, low carbohydrate':ab,ti

#5. 'carbohydrate diets, low':ab,ti

#4. 'carbohydrate diet, low':ab,ti

#3. 'diet, low carbohydrate':ab,ti

#2. 'diet, carbohydrate restricted':ab,ti

#1. 'low carbohydrate diet'/exp

Cochrane Library Trials

#1 MeSH descriptor: [Diet, Carbohydrate-Restricted] explode all trees

#2 (Diet, Carbohydrate Restricted):ti,ab,kw (Word variations have been searched)

#3 (Diet, Carbohydrate Restricted):ti,ab,kw (Word variations have been searched)

#4 (Carbohydrate Diet, Low):ti,ab,kw (Word variations have been searched)

#5 (Low Carbohydrate Diets):ti,ab,kw (Word variations have been searched)

#6 (Carbohydrate-Restricted Diet):ti,ab,kw (Word variations have been searched)

#7 (Carbohydrate-Restricted Diet):ti,ab,kw (Word variations have been searched)

#8 (Carbohydrate-Restricted Diets):ti,ab,kw (Word variations have been searched)

#9 (Diets, Carbohydrate-Restricted):ti,ab,kw (Word variations have been searched)

#10 (Low-Carbohydrate Diet):ti,ab,kw (Word variations have been searched)

#11 (Diet, Low-Carbohydrate):ti,ab,kw (Word variations have been searched)

#12 (Diets, Low-Carbohydrate):ti,ab,kw (Word variations have been searched)

#13 (Low Carbohydrate Diet):ti,ab,kw (Word variations have been searched)

#14 (Low-Carbohydrate Diets):ti,ab,kw (Word variations have been searched)

#15 (Low-Carbohydrate Diet):ti,ab,kw (Word variations have been searched)

#16 (Atkins Diet):ti,ab,kw (Word variations have been searched)

#17 (Diet, Atkins):ti,ab,kw (Word variations have been searched)

#18 #1 or #2 or #3 or #4 or #5 or #6 or #7 or #8 or #9 or #10 or #11 or #12 or #13 or #14 or #15 or #16 or #17

#19 MeSH descriptor: [Cardiovascular Diseases] explode all trees

#20 (Disease, Cardiovascular):ti,ab,kw (Word variations have been searched)

#21 (Diseases, Cardiovascular):ti,ab,kw (Word variations have been searched)

#22 #19 or #20 or #21

#23 #18 and #22
