# Supplementary material for: The effects of low-carbohydrate diets on cardiovascular risk factors: A meta-analysis
Source: PLoS One. 2020 Jan 14;15(1):e0225348. doi: 10.1371/journal.pone.0225348 (PMC6959586; doi:10.1371/journal.pone.0225348)
Supplement: S3 Table — (DOCX) [file pone.0225348.s014.docx]

**S2 Table excluded studies and reasons for exclusion**

| Reasons for exclusion | Number of  studies excluded |
| --- | --- |
| Duration of the intervention <12 weeks | Frank[48]  Jillon[49] |
| Not a randomised controlled trial | Jeannie[50] |
| No original data | Boris[51]  Sondike[52] |
| Treatment and control of carbohydrates  are similar - not a qualified comparison | Clar[53] |
| Treatment diet is not low in carbohydrates | Peter[54]  Cheryl[55] |
| Less than 10 participants randomised per group | Jorgen[56] |
| Inconsistent interventions | Jessica[57] |
| No required detection indicators | Nichola[58] |
| Could not obtain full text | Thomas[59] |
