# Supplementary material for: The effects of low-carbohydrate diets on cardiovascular risk factors: A meta-analysis
Source: PLoS One. 2020 Jan 14;15(1):e0225348. doi: 10.1371/journal.pone.0225348 (PMC6959586; doi:10.1371/journal.pone.0225348)
Supplement: S4 Table — (DOCX) [file pone.0225348.s015.docx]

**S3 Table.The main indicators observed in each study**

| Author | Year | Main outcome mesurse |
| --- | --- | --- |
| Gary | 2010 | [triglyceride 、LDL、HDL、weight、systolic blood pressure、diastolic blood pressure](file:///D:\\Program%20Files%20(x86)\\Youdao\\Dict\\8.0.0.0\\resultui\\html\\index.html" \l "javascript:;" \o "file:///D:Program Files (x86)YoudaoDict8.0.0.0resultuihtmlindex.html#javascript:;) |
| Morgan | 2008 | [triglyceride 、LDL、HDL、weight、fasting plasma glucose](file:///D:\\Program%20Files%20(x86)\\Youdao\\Dict\\8.0.0.0\\resultui\\html\\index.html" \l "javascript:;" \o "file:///D:Program Files (x86)YoudaoDict8.0.0.0resultuihtmlindex.html#javascript:;) |
| Lydia | 2014 | [triglyceride 、LDL、HDL、weight、systolic blood pressure](file:///D:\\Program%20Files%20(x86)\\Youdao\\Dict\\8.0.0.0\\resultui\\html\\index.html" \l "javascript:;" \o "file:///D:Program Files (x86)YoudaoDict8.0.0.0resultuihtmlindex.html#javascript:;) |
| Lean | 1996 | TC、triglyceride 、LDL、HDL、  weight、systolic blood pressure、diastolic blood pressure |
| Frank | 2009 | TC、triglyceride 、LDL、HDL、fasting plasma glucose  systolic blood pressure、diastolic blood pressure |
| Elhayany | 2010 | TC、triglyceride 、LDL、HDL、weight、fasting plasma glucose |
| Jeannie | 2015 | TC、triglyceride 、LDL、HDL、fasting plasma glucose |
| Gary | 2003 | TC、triglyceride 、LDL、HDL、weight  systolic blood pressure、diastolic blood pressure |
| Bonnie | 2003 | TC、triglyceride 、LDL、HDL、fasting plasma glucose  systolic blood pressure、diastolic blood pressure |
| Jeannie | 2017 | TC、triglyceride 、LDL、HDL、weight、fasting plasma glucose  systolic blood pressure、diastolic blood pressure |
| Xin Liu | 2013 | TC、triglyceride 、LDL、HDL、weight、fasting plasma glucose 、systolic blood pressure、diastolic blood pressure |
| Lim | 2010 | TC、triglyceride 、LDL、HDL、weight、fasting plasma glucose  systolic blood pressure、diastolic blood pressure |
