# Supplementary material for: The effects of low-carbohydrate diets on cardiovascular risk factors: A meta-analysis
Source: PLoS One. 2020 Jan 14;15(1):e0225348. doi: 10.1371/journal.pone.0225348 (PMC6959586; doi:10.1371/journal.pone.0225348)
Supplement: S5 Table — (DOCX) [file pone.0225348.s016.docx]

**S4 Table.General characteristics of included literature**

| Author | Year | Parallel design | Number of patients | Man/  Female | Age | BMI | C  ountry | Duration of follow-up | Low-carbohydrate diet |
| --- | --- | --- | --- | --- | --- | --- | --- | --- | --- |
| Gary[15] | 2010 | Yes | 307 | 99/208 | 45.5±9.7 | 36.1±3.5 | USA | 2 years | carbohydrate intake of at most 20 g |
| Morgan[16] | 2008 | Yes | 118 | 30/88 | 40.85±9.65 | 31.7±2.55 | UK | 6month | Aktin Diet |
| Lydia  [17] | 2014 | Yes | 148 | 31/131 | 46.8±10.15 | 35.4±4.15 | USA | 6month | (total carbohydrate minus total fiber) |
|  |  |  |  |  |  |  |  |  | of less than 40 g/d |
| Lean  [18] | 1996 | Yes | 110 | 0/110 | 50.6±13.8 | 32.55±5.3 | UK | 6month | Carbohydrates corresponding to 35% of |
|  |  |  |  |  |  |  |  |  | energy intake |
| Frank  [19] | 2009 | Yes | 403 | 139/264 | 50.5±9.5 | 33±4 | USA | 2year | Carbohydrates corresponding to 35% of |
|  |  |  |  |  |  |  |  |  | energy intake |
| Elhayany  [20] | 2010 | Yes | 124 | 66/58 | 56.3±6.2 | 31.4±3 | Israel | 12months | Carbohydrates corresponding to 35% of |
|  |  |  |  |  |  |  |  |  | energy intake |
| Jeannie  [21] | 2015 | Yes | 115 | 66/49 | 58±7 | 34.6±4.3 | Australia | 52weeks | 14% of energy as carbohydrate |
|  |  |  |  |  |  |  |  |  | (carbohydrate ,50 g/d) |
| Gary  [22] | 2003 | Yes | 63 | 20/43 | 44.1±8.2 | 34.2±5.4 | USA | 1year | Aktin Diet |
| Bonnie  [23] | 2003 | Yes | 42 | NA | 43.7±7.7 | 37.65±1.83 | USA | 6months | carbohydrate intake of at most 20 g |
| Jeannie  [24] | 2017 | Yes | 115 | 66/49 | 58±7 | 34.6±4.3 | Australia | 2 years | 14% carbohydrate(<50g/d) |
| Xin Liu  [25] | 2013 | Yes | 49 | NA | 47.9±0.9 | 26.7±0.3 | China | 12 weeks | initial carbohydrateintake 20 g/d, with a 10 g |
|  |  |  |  |  |  |  |  |  | increase weekly |
| Lim  [26] | 2010 | Yes | 46 | NA | 47±10 | 32±6 | Australia | 15months | 4%carbohydrate |
