# Supplementary material for: The effects of low-carbohydrate diets on cardiovascular risk factors: A meta-analysis
Source: PLoS One. 2020 Jan 14;15(1):e0225348. doi: 10.1371/journal.pone.0225348 (PMC6959586; doi:10.1371/journal.pone.0225348)
Supplement: S6 Table — (DOCX) [file pone.0225348.s017.docx]

**S3.Table Subgroup analysis of major cardiovascular risk factors Triglyceride**

| subgroup | No.of studies | MD(95%CI) | P for heterogeneity | I^2^(%) |
| --- | --- | --- | --- | --- |
| state |  |  |  |  |
| America | 5 | -0.09(-0.17,0.00) | 0.34 | 12 |
| Australia | 3 | -0.33(-0.48,-0.17) | 0.69 | 0 |
| England | 2 | -0.18(-0.57,0.20) | 0.01 | 84 |
| China | 1 | -0.43(-0.90,0.04) |  |  |
| Isrel | 1 | 0.08(-0.04,0.20) |  |  |
| Age,year |  |  |  |  |
| ＜50 | 5 | -0.02(-0.10,0.06) | 0.01 | 68 |
| ≥50 | 7 | -0.17(-0.26,-0.09) | 0.08 | 47 |
| samples |  |  |  |  |
| ＜100 | 4 | -0.18(-0.30,-0.06) | 0.57 | 0 |
| ≥100 | 8 | -0.07(-0.14,-0.01) | <0.001 | 73 |
