# Supplementary material for: The effects of low-carbohydrate diets on cardiovascular risk factors: A meta-analysis
Source: PLoS One. 2020 Jan 14;15(1):e0225348. doi: 10.1371/journal.pone.0225348 (PMC6959586; doi:10.1371/journal.pone.0225348)
Supplement: S7 Table — (DOCX) [file pone.0225348.s018.docx]

**S6.Table Subgroup analysis of major cardiovascular risk factors HDL**

| subgroup | No.of studies | MD(95%CI) | P for heterogeneity | I^2^(%) |
| --- | --- | --- | --- | --- |
| state |  |  |  |  |
| America | 5 | 0.09(0.05,0.13) | 0.39 | 4 |
| Australia | 3 | 0.08(0.01,0.16) | 0.43 | 0 |
| England | 2 | 0.09(0.02,0.16) | 0.67 | 0 |
| China | 1 | 0.30(0.09,0.51) |  |  |
| Isrel | 1 | 0.12(0.05,0.19) |  |  |
| Age,year |  |  |  |  |
| ＜50 | 7 | 0.11（0.06,0.16) | 0.29 | 18 |
| ≥50 | 5 | 0.09(0.05,0.13) | 0.64 | 0 |
| samples |  |  |  |  |
| ＜100 | 4 | 0.16（0.07,0.24) | 0.16 | 42 |
| ≥100 | 8 | 0.09(0.06,0.12) | 0.9 | 0 |
