# Supplementary material for: The effects of low-carbohydrate diets on cardiovascular risk factors: A meta-analysis
Source: PLoS One. 2020 Jan 14;15(1):e0225348. doi: 10.1371/journal.pone.0225348 (PMC6959586; doi:10.1371/journal.pone.0225348)
Supplement: S8 Table — (DOCX) [file pone.0225348.s019.docx]

**S7 Table.Subgroup analysis of major cardiovascular risk factors LDL**

| subgroup | No.of studies | MD(95%CI) | P for heterogeneity | I^2^(%) |
| --- | --- | --- | --- | --- |
| state |  |  |  |  |
| America | 5 | 0.09(0.01,0.18) | 0.91 | 0 |
| Australia | 3 | 0.08(-0.13,0.28) | 0.92 | 0 |
| England | 2 | 0.06(-0.14,0.27) | 0.54 | 0 |
| China | 1 | 0.55(0.02,1.08) |  |  |
| Isrel | 1 | -0.17(-0.41,0.07) |  |  |
| Age,year |  |  |  |  |
| ＜50 | 5 | 0.04(-0.07,0.15) | 0.45 | 0 |
| ≥50 | 7 | 0.09(0.00,0.19) | 0.63 | 0 |
| samples |  |  |  |  |
| ＜100 | 4 | 0.14(-0.00,0.28) | 0.36 | 6 |
| ≥100 | 8 | 0.05(-0.03,0.13) | 0.75 | 0 |
