# Supplementary material for: The effects of low-carbohydrate diets on cardiovascular risk factors: A meta-analysis
Source: PLoS One. 2020 Jan 14;15(1):e0225348. doi: 10.1371/journal.pone.0225348 (PMC6959586; doi:10.1371/journal.pone.0225348)
Supplement: S9 Table — (DOCX) [file pone.0225348.s020.docx]

**S9 Table Subgroup analysis of major cardiovascular risk factors Weight**

| subgroup | No.of studies | MD(95%CI) | P for heterogeneity | I^2^(%) |
| --- | --- | --- | --- | --- |
| state |  |  |  |  |
| America | 3 | -1.36(-2.57,-0.15) | 0.26 | 26 |
| England | 4 | -1.27(-2.68,0.14) | 0.3 | 18 |
| China | 1 | -0.20(-1.05,0.65) |  |  |
| Isrel | 1 | -1.50(-5.60,2.60) |  |  |
| Age,year |  |  |  |  |
| ＜50 | 6 | -0.70(-1.37,-0.03) | 0.1 | 45 |
| ≥50 | 3 | -0.95(-2.53,0.63) | 0.83 | 0 |
| samples |  |  |  |  |
| ＜100 | 3 | -0.35(-1.14,0.44) | 0.58 | 0 |
| ≥100 | 6 | -1.34(-2.32,-0.35) | 0.29 | 18 |
